# Nuclear focal adhesion kinase induces APC/C activator protein CDH1-mediated cyclin-dependent kinase 4/6 degradation and inhibits melanoma proliferation

**DOI:** 10.1016/j.jbc.2022.102013

**Published:** 2022-05-05

**Authors:** James M. Murphy, Kyuho Jeong, Eun-Young Erin Ahn, Ssang-Taek Steve Lim

**Affiliations:** 1 Department of Pathology, University of Alabama at Birmingham, Birmingham, Alabama, USA; 2 Department of Biochemistry and Molecular Biology, College of Medicine, University of South Alabama, Mobile, Alabama, USA; 3 Department of Pathology, O'Neal Comprehensive Cancer Center, University of Alabama at Birmingham, Birmingham, Alabama, USA

**Keywords:** FAK, CDK4/6, CDH1, melanoma, APC/C, anaphase-promoting complex/cyclosome, CDH1, CDC homolog 1, CDK, cyclin-dependent kinase, CDKI, CDK inhibitor, FAK, focal adhesion kinase, FAK-I, FAK inhibitor, FRNK, FAK-related nonkinase, GST, glutathione-*S*-transferase, HEK 293T, human embryonic kidney 293T cell line, IP, immunoprecipitate, KD, kinase dead, MEK, mitogen-activated protein kinase/extracellular signal–regulated kinase kinase, NLM, nonnuclear-localizing mutant, PCNA, proliferating cell nuclear antigen, PD-1, programmed cell death protein 1, PD-L1, programmed death–ligand 1, pRb, retinoblastoma protein, pY397, autophosphorylation at tyrosine 397, qPCR, quantitative PCR

## Abstract

Dysregulation of cyclin-dependent kinases (CDKs) can promote unchecked cell proliferation and cancer progression. Although focal adhesion kinase (FAK) contributes to regulating cell cycle progression, the exact molecular mechanism remains unclear. Here, we found that FAK plays a key role in cell cycle progression potentially through regulation of CDK4/6 protein expression. We show that FAK inhibition increased its nuclear localization and induced G1 arrest in B16F10 melanoma cells. Mechanistically, we demonstrate nuclear FAK associated with CDK4/6 and promoted their ubiquitination and proteasomal degradation through recruitment of CDC homolog 1 (CDH1), an activator and substrate recognition subunit of the anaphase-promoting complex/cyclosome E3 ligase complex. We found the FAK N-terminal FERM domain acts as a scaffold to bring CDK4/6 and CDH1 within close proximity. However, overexpression of nonnuclear-localizing mutant FAK FERM failed to function as a scaffold for CDK4/6 and CDH1. Furthermore, shRNA knockdown of CDH1 increased CDK4/6 protein expression and blocked FAK inhibitor–induced reduction of CDK4/6 in B16F10 cells. *In vivo*, we show that pharmacological FAK inhibition reduced B16F10 tumor size, correlating with increased FAK nuclear localization and decreased CDK4/6 expression compared with vehicle controls. In patient-matched healthy skin and melanoma biopsies, we found FAK was mostly inactive and nuclear localized in healthy skin, whereas melanoma lesions showed increased active cytoplasmic FAK and elevated CDK4 expression. Taken together, our data demonstrate that FAK inhibition blocks tumor proliferation by inducing G1 arrest, in part through decreased CDK4/6 protein stability by nuclear FAK.

Melanoma accounts for nearly all skin cancer–related deaths, even though melanoma constitutes a small fraction of total skin cancer cases. While the 5-year survival rate for melanoma across all stages is approximately 93%, nonoperable and metastatic melanoma have a 5-year survival rate around 27% (1). Several therapies have been approved by the Food and Drug Administration to treat patients with nonoperable or metastatic melanoma including BRAF inhibitors and immune checkpoint therapies. Immune checkpoint therapies, such as antibodies targeting programmed cell death protein 1 (PD-1) and programmed death–ligand 1 (PD-L1), prevent tumor cells from evading cytotoxic T cells leading to their clearance by the immune system and have shown efficacy in treating advanced melanoma (2, 3, 4). Recent studies have demonstrated that using anti-PD-1 therapy in combination with anti–cytotoxic T lymphocyte–associated antigen 4 therapy significantly improves metastatic melanoma patient survival outcome (5, 6, 7). This combination is thought to increase the immune response against tumors by increasing cytotoxic T-cell activation (5). Currently, anti-PD-1 therapy is being investigated in combination with other immunotherapies or treatment strategies (*i.e.*, BRAF, mitogen-activated protein kinase/extracellular signal–regulated kinase kinase [MEK] inhibitors) in clinical trials (reviewed in Ref. (8)). While BRAF inhibitors are the most common treatment for melanoma, their use is also limited to the approximately 40% of patients that harbor an activating BRAF mutation (9). However, combinatorial BRAF/MEK inhibition has become the standard treatment for melanoma patients. Recent studies have shown that cotreatment with BRAF/MEK inhibitors not only improves patient outcome compared with monotherapy but also reduces drug side effects (10). Unfortunately, patients who develop BRAF inhibitor resistance or had previously undergone BRAF inhibitor treatment show resistance to MEK inhibitors (11). As malignant melanoma is still fatal despite these therapies, new clinically active treatments are needed to overcome these limitations or possibly enhance existing therapies in treating melanoma.

Hyperactivation of the cyclin-dependent kinase 4/6 (CDK4/6)-pRb-p16INK4A pathway has been reported in approximately 90% of melanomas (12, 13, 14). Amplification of D-type cyclins and CDK4/6, loss of p16INK4A expression, or mutations in either p16INK4A or CDK4 that disrupt their association result in elevated CDK4/6 activity and cell cycle progression (15, 16, 17, 18). CDK4/6 are activated through their interaction with D-type cyclins, which allow CDK4/6-mediated phosphorylation of pRb. Phosphorylated pRb then releases E2F transcription factor, thus promoting expression of genes necessary for cell cycle progression. As such, identification of CDK inhibitors (CDKIs) to block cell cycle progression in tumor cells has gained a lot of attention. Although there were several concerns regarding nonspecific nature and side effects of first-generation CDKIs (19, 20), significantly improved CDKIs that target specific CDKs are undergoing clinical trials. Currently, the only CDKIs that have been approved for clinical use target CDK4/6 in breast cancer patients (21, 22). The efficacy of CDKIs is still being tested in phase I and II clinical trials for their use to treat other cancers, including melanoma, small cell lung cancer, colon cancer, ovarian cancer, and lymphoma.

Focal adhesion kinase (FAK) is a protein tyrosine kinase commonly overexpressed in advanced human cancers. FAK primarily mediates signaling cascades downstream of integrin, growth factor, cytokine, and G protein–coupled receptors at the cell surface to promote cell proliferation and migration. FAK signaling promotes important malignant features in cancer cells, such as cancer stemness, epithelial-to-mesenchymal transition, tumorigenesis, chemotherapeutic resistance, and stromal fibrosis (23, 24, 25). As such, several small-molecule FAK inhibitors (FAK-Is) are currently being tested in clinical trials as anticancer agents (23). Interestingly, our recent work found that nuclear FAK also plays an important role in the regulation of several nuclear factors in cell cycle progression. In smooth muscle cells, it was shown that nuclear FAK blocked cell proliferation through decreased stability of the GATA4 transcription factor, which reduced cyclin D1 transcription and induced cell cycle arrest (26). In addition, nuclear FAK can block cell cycle progression in smooth muscle cells by increasing protein expression of CDKI proteins p21/p27 through loss of S-phase kinase–associated protein 2 (Skp2) (27). However, it remains to be determined if nuclear FAK controls proliferation in cancer cells.

In this study, we investigated the signaling role of both FAK activity and its cellular localization in cell cycle regulation and cancer progression in B16F10 melanoma. We chose B16F10 melanoma as they have a large deletion within the *CDKN2A* locus (encoding p16INK4A) and increased CDK4/6 activity (28). We found that FAK inhibition induced G1-cell cycle arrest, and this was associated with decreased CDK4/6 protein expression. Mechanistically, FAK inhibition promoted FAK nuclear localization, where it promoted CDK4/6 ubiquitination and proteasomal degradation *via* recruitment of CDC homolog 1 (CDH1), an activator of the anaphase-promoting complex/cyclosome (APC/C) E3 ligase complex. In addition, in a B16F10 flank tumor model, pharmacological FAK inhibition reduced tumor size, increased FAK nuclear localization, and reduced CDK4/6 protein expression. Interestingly, we found that FAK exhibited increased activity and cytoplasmic localization, which was correlated with increased CDK4 expression, in human melanoma compared with same-patient normal skin controls. Overall, our data demonstrate the potential for FAK-Is to reduce melanoma growth through forced nuclear localization of FAK to regulate CDK4/6 expression.

## Results

### FAK inhibition induces G1 arrest potentially through loss of CDK4/6

**Figure 1:**
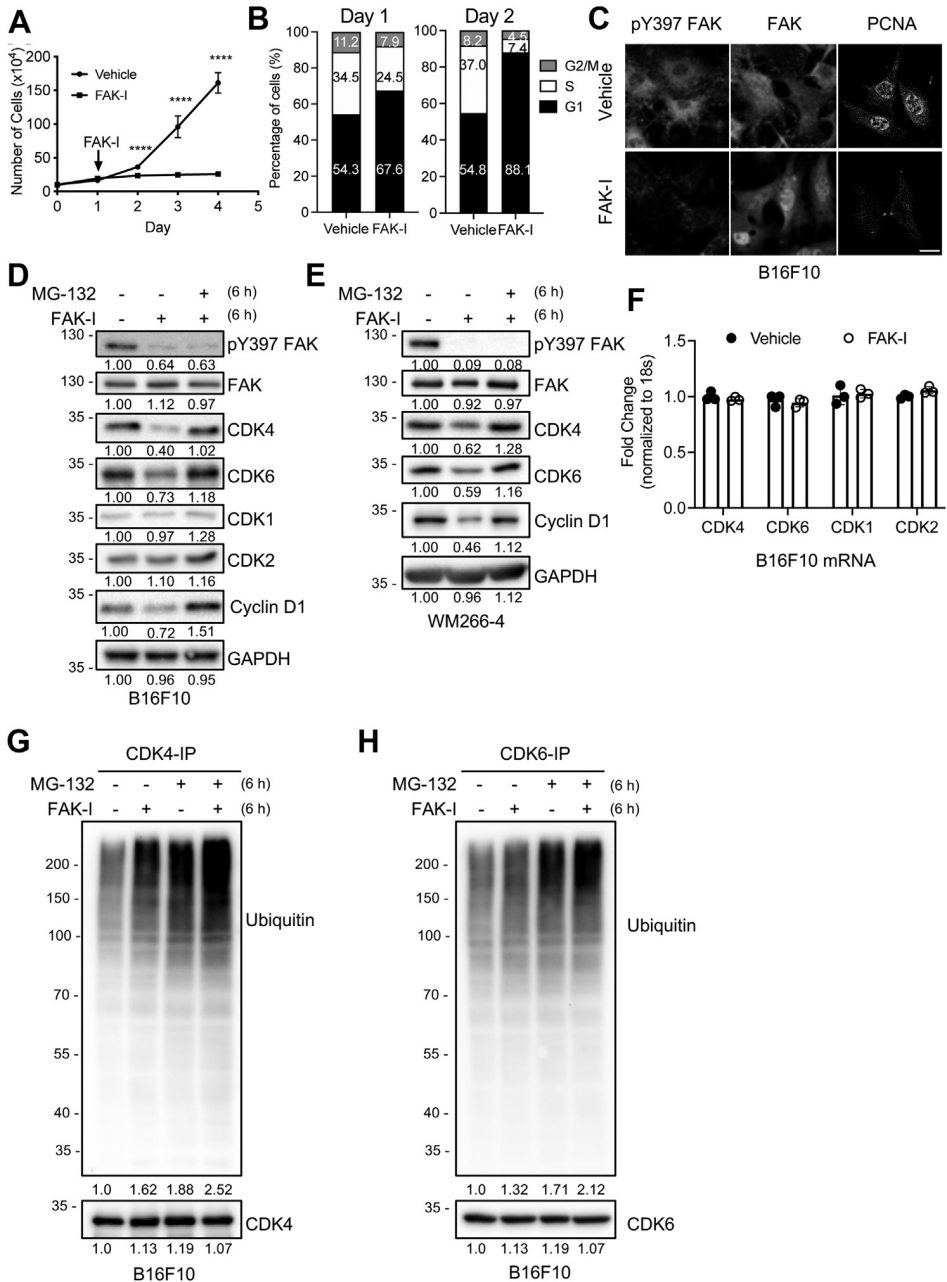
**FAK inhibition slows B16F10 proliferation through accelerated turnover of CDK4/6, resulting in G1 cell cycle arrest.** B16F10 cells were treated with either vehicle or FAK-I (PF-271, 2.5 μM) for indicated times. *A*, viable cells were enumerated each day and plotted for 4 days (n = 3, ±SD). ∗∗∗∗*p* < 0.001. *B*, cell cycle analysis was performed, and percentage of cells in G1, S, or G2/M phase is shown (n = 3). *C*, immunostaining for FAK, pY397 FAK and PCNA following 24 h treatment with either vehicle or FAK-I (n = 3). The scale bar represents 20 μm. *D*, FAK inhibition promoted proteasomal degradation of CDK4/6 but not CDK1/2. B16F10 cells were treated with FAK-I with or without a proteasome inhibitor (MG-132, 10 μM) for 6 h. Immunoblots were performed for pY397 FAK, FAK, CDK4, CDK6, CDK1, CDK2, cyclin D1, and GAPDH as loading control. Protein expression was normalized to GAPDH, and fold change to untreated is shown (n = 3). *E*, WM266-4 human melanoma cells were treated with FAK-I with or without a proteasome inhibitor (MG-132, 10 μM) for 6 h. Immunoblots were performed for pY397 FAK, FAK, CDK4, CDK6, cyclin D1, and GAPDH as loading control. Protein expression was normalized to GAPDH, and fold change to untreated is shown (n = 3). *F*, FAK inhibition did not alter CDK4 and CDK6 mRNA expression as determined by RT–qPCR (n = 3). *G* and *H*, FAK inhibition increased ubiquitination of CDK4/6 in B16F10. B16F10 cells were treated with combinations of FAK-I (2.5 μM) and MG-132 (10 μM) for 6 h. Immunoblots of (*G*) CDK4 immunoprecipitates (IPs) or (*H*) CDK6-IP for ubiquitin, and CDK4 or CDK6 as loading control. Total ubiquitinated proteins were normalized to CDK4 or CDK6, and fold change over untreated is shown (n = 3). CDK, cyclin-dependent kinase; FAK, focal adhesion kinase; FAK-I, FAK inhibitor; PCNA, proliferating cell nuclear antigen; pY397, autophosphorylation at tyrosine 397; qPCR, quantitative PCR.

As FAK activity has been shown to be important for cell proliferation, we first examined if FAK inhibition blocked proliferation of B16F10 melanoma cells. While vehicle-treated cells showed robust proliferation over 4 days, cells treated with a FAK-I (PF-271) showed little to no proliferation over 4 days (Fig. 1*A*). To get a better understanding of how FAK inhibition could reduce B16F10 proliferation, we performed cell cycle analysis after 24 and 48 h FAK inhibition. FAK-I increased the percentage of cells in G1 phase at both 24 and 48 h compared with vehicle treated (Fig. 1*B*). Furthermore, vehicle-treated cells showed high levels of proliferating cell nuclear antigen (PCNA), but cells treated with FAK-I showed decreased PCNA immunostaining (Fig. 1*C*). Increased PCNA levels were associated with high levels of cytoplasmic FAK and active pY397 (autophosphorylation at tyrosine 397) FAK (Fig. 1*C*). However, B16F10 cells treated with FAK-I showed increased FAK nuclear localization and reduced active pY397 FAK staining (Fig. 1*C*). The results suggested that inactive nuclear FAK may reduce B16F10 cell proliferation, through an unknown mechanism.

As nuclear FAK is known to act as a scaffold for the ubiquitination and degradation of several nuclear factors (26, 27, 30, 31), we analyzed our own and publicly available mass spectrometry data to identify possible nuclear FAK-interacting proteins involved in cell cycle progression. Potential candidates included CDK4, CDK9, cyclin D1, cyclin T1, and p21 (29). Of these, CDK4 plays a key role in promoting G1-to-S progression through inhibitory phosphorylation of retinoblastoma protein (pRb). B16F10 cells treated with FAK-I for 6 h showed decreased CDK4 protein expression (Fig. 1*D*). FAK-I also reduced CDK4 protein expression and cell proliferation in multiple human melanoma cell lines (Fig. 1*E*, Supporting Data 1 and 2), showing this effect was not specific to murine melanoma. To see if this was unique to CDK4, we also examined if FAK inhibition reduced expression of other CDKs. Interestingly, while FAK-I was also able to reduce CDK6 protein, it had no effect on CDK1/2 protein expression in B16F10 cells (Fig. 1*D*). Similarly, CDK6 protein expression was reduced by FAK-I treatment in the human melanoma cell lines (Fig. 1*E* and Supporting Data 1). To test post-translational regulation of CDK4/6 protein expression, we cotreated cells with the proteasomal inhibitor MG-132. Treatment with MG-132 was able to rescue CDK4/6 protein expression in both murine and human melanoma cells (Fig. 1, *D* and *E* and Supporting Data 1). However, cotreatment with MG-132 had no effect on CDK1/2 (Fig. 1*D*), supporting FAK regulation of CDK4/6 stability but not CDK1/2. Analysis of CDK4/6 and CDK1/2 mRNA expression showed that FAK-I indeed had no effect on their transcription (Fig. 1*F*), supporting a role for FAK-mediated post-translational regulation of CDK4/6 protein. Decreased CDK4/6 expression was also associated with loss of pRb phosphorylation (Supporting Data 3A), further supporting that cell cycle progression is blocked by FAK inhibition. As it has been shown that mutant cyclin D1 (K114E) has a shorter half-life because of its inability to bind CDK4 (32), we examined the effect of FAK-I on D-type cyclin expression. Interestingly, FAK inhibition for 6 h reduced protein expression, but not mRNA, of all three D-type cyclins in B16F10 and WM266-4 cells (Fig. 1, *D* and *E* and Supporting Data 3, *A* and *B*). However, as we have previously showed FAK inhibition can reduce GATA4-mediated cyclin D1 transcription (26), we evaluated if FAK-I reduced D-type cyclin mRNA at later time points. After 24 and 48 h, FAK-I treatment was able to reduce mRNA expression of all three D-type cyclins (Supporting Data 3, *C*–*E*). CDK4 mRNA was unchanged after 24 and 48 h of FAK-I treatment (Supporting Data 3*F*), further supporting that FAK may regulate CDK4/6 protein stability. While we saw reduced GATA4 protein but no change in D-type cyclin mRNA at 6 h (Supporting Data 3, *A* and *B*), the early loss of D-type cyclin protein expression could be through a FAK-independent mechanism. Overexpression of cyclin D1 in human embryonic kidney 293T (HEK 293T) cells showed that early FAK-I treatment could increase cyclin D1 ubiquitination (Supporting Data 3*G*); this could be due to the decreased half-life cyclin D1 exhibits when not bound to CDK4/6. These data show that FAK inhibition induced G1 arrest, in part through increased degradation of CDK4/6 resulting in loss of pRb phosphorylation.

### FAK promotes CDK4/6 degradation *via* N-terminal FERM domain

**Figure 2:**
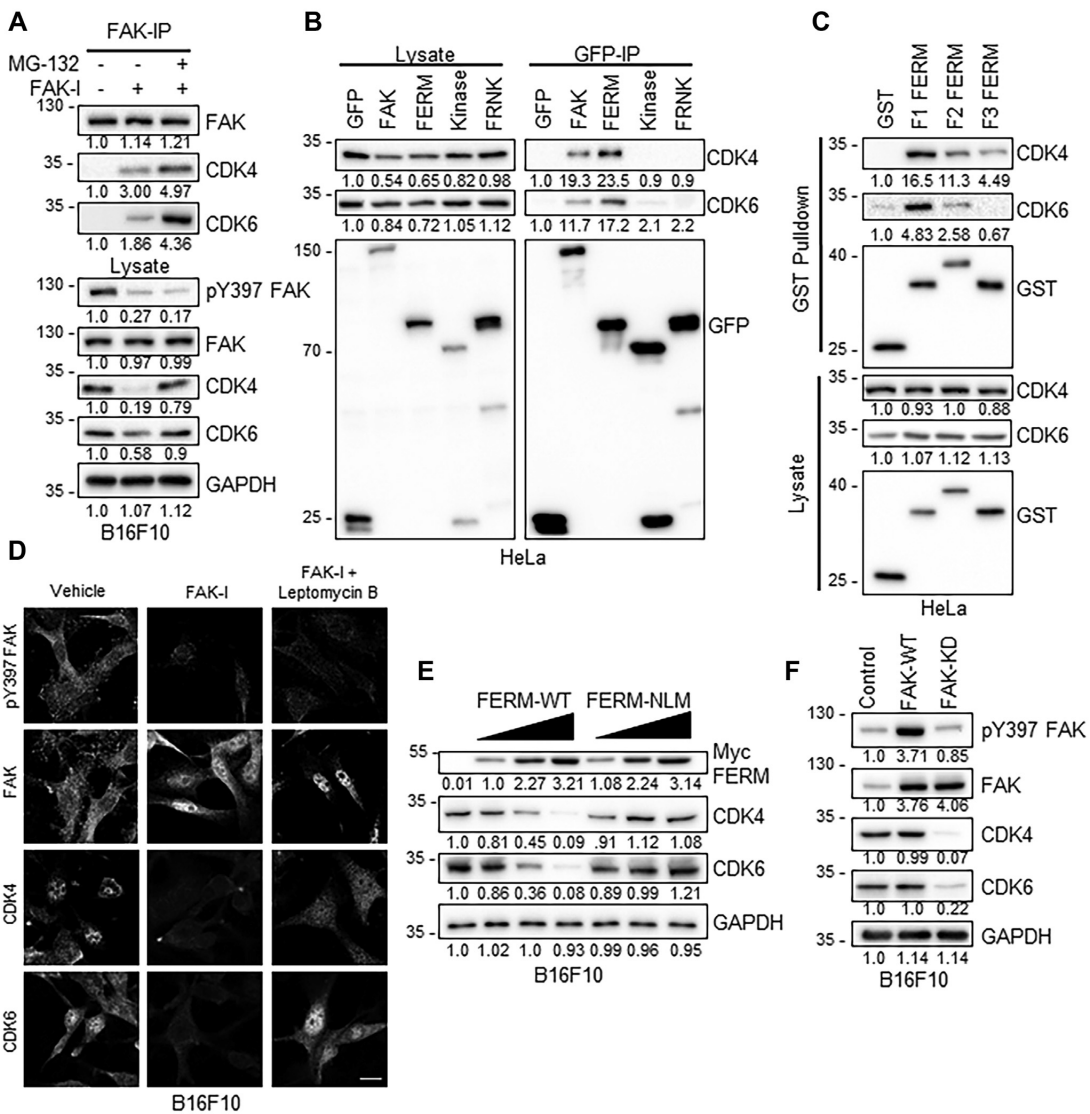
**FAK FERM promotes proteasomal degradation of CDK4/6.***A*, FAK inhibition promotes FAK–CDK4/6 association and is enhanced by cotreatment with MG-132. B16F10 cells were treated with FAK-I (PF-271, 2.5 μM) with or without MG-132 (10 μM) for 6 h (n = 3). Immunoblotting of FAK-immunoprecipitates (IPs; *top panel*) was performed for FAK, CDK4, and CDK6. Total lysates (*low panel*) were probed for pY397 FAK, FAK, CDK4, CDK6, and GAPDH. Protein expression was normalized to either FAK (in FAK IP) or GAPDH (in lysate blots), and fold change to untreated is shown (n = 3). *B*, HeLa cells were transfected with GFP, GFP-tagged FAK, FERM, kinase, or FRNK constructs and treated with FAK-I and MG-132 for 6 h prior to immunoprecipitation. CDK4/6 associate with the N-terminal FAK-FERM domain. Immunoblotting of lysates and GFP-IP was performed for GFP, CDK4, and CDK6. Protein expression was normalized to either GFP (in IP) or GAPDH (in lysate), and fold change to GFP lane is shown (n = 3). *C*, CDK4/6 preferentially associate with the F1 and F2 FERM domains. HeLa cells were transfected with GST, GST-tagged F1, F2, or F3 FERM constructs. Immunoblotting of lysates and GST pulldown for GST, CDK4, and CDK6. Protein expression was normalized to either GST (in pulldown) or GAPDH (in lysate), and fold change to GST lane is shown (n = 3). *D*, FAK-I-induced CDK4/6 degradation occurs in the cytoplasm. B16F10 cells were treated with FAK-I with or without leptomycin B for 6 h. Immunostaining for FAK, pY397 FAK, CDK4, and CDK6 is shown (n = 3). The scale bar represents 20 μm. *E*, nuclear localization of the FAK-FERM domain is required for CDK4/6 degradation. B16F10 cells were transduced with increasing MOI of Myc-FERM-WT or Myc-FERM-NLM adenovirus for 24 h. Immunoblotting of total lysates for CDK4, CDK6, Myc, and GAPDH as loading control. Protein expression was normalized to GAPDH, and fold change to nontransduced is shown (n = 3). *F*, FAK-KD (kinase-dead) reduces CDK4/6 expression. B16F10 cells were transduced with FAK-WT or FAK-KD adenovirus. Immunoblotting of total lysates for pY397 FAK, FAK, CDK4, CDK6, and GAPDH as loading control. Protein expression was normalized to GAPDH, and fold change to nontransduced is shown (n = 3). CDK, cyclin-dependent kinase; FAK, focal adhesion kinase; FAK-I, FAK inhibitor; FRNK, FAK-related nonkinase; GST, glutathione-*S*-transferase; MOI, multiplicity of infection; NLM, nonnuclear-localizing mutant; pY397, autophosphorylation at tyrosine 397.

To better understand how FAK could regulate CDK4/6 protein stability, we examined whether FAK-I increased ubiquitination of CDK4/6 in B16F10 melanoma cells. Immunoblotting of CDK4 and CDK6 immunoprecipitates (IPs) showed increased polyubiquitinated bands following FAK-I treatment compared with vehicle treated (Fig. 1, *G* and *H*). Cotreatment with FAK-I and MG-132 showed even higher levels of ubiquitination compared with FAK-I or MG-132 alone (Fig. 1, *G* and *H*). As nuclear FAK is known to regulate stability of several nuclear factors *via* ubiquitination, we next tested to see if FAK could interact with CDK4/6 in B16F10 cells. While we were unable to detect CDK4/6 interaction in FAK-IP of vehicle-treated cells, we saw FAK–CDK4/6 interaction following FAK inhibition (Fig. 2*A*). This association was further increased by cotreatment with MG-132 (Fig. 2*A*). To identify which FAK domain associated with CDK4/6, we overexpressed GFP fusion constructs for either full-length FAK, N-terminal FERM, kinase, or C-terminal FAK-related nonkinase (FRNK) domain in HeLa cells (Supporting Data 4*A*) (31). Immunoblotting of GFP-IPs demonstrated that CDK4/6 interacted with both full-length FAK and the FERM domain (Fig. 2*B*). Furthermore, we observed that CDK4/6 levels were lower in the lysates of cells transfected with FAK and FERM (Fig. 2*B*), supporting the notion that FAK FERM domain binds to CDK4/6 to regulate their stability. The FERM domain is comprised of three distinct lobes: F1, F2, and F3. To identify which lobe interacts with CDK4/6, we transfected HeLa cells with glutathione-*S*-transferase (GST)-FERM F1, F2, or F3 constructs (Supporting Data 4*B*) (31). GST pulldown revealed that CDK4/6 most strongly interacted with the F1 lobe of the FERM domain and to a lesser extent the F2 lobe (Fig. 2*C*). These findings are consistent with our previous work demonstrating that the F1 and F2 lobes of the FAK-FERM domain recruit nuclear factors for degradation (31). To test if CDK4/6 degradation occurs in the nucleus or cytoplasm, we treated B16F10 cells with FAK-I in combination with the nuclear export blocker leptomycin B. Immunostaining revealed that FAK-I alone could increase FAK nuclear localization and decrease pY397 FAK levels after 6 h (Fig. 2*D*). Increased nuclear FAK was associated with decreased nuclear staining of CDK4/6 in FAK-I-treated cells (Fig. 2*D*). While FAK-I cotreatment with leptomycin B further increased FAK nuclear localization, it completely blocked FAK-I-mediated loss of CDK4/6 from the nucleus (Fig. 2*D*). To determine if increased CDK4/6 nuclear localization following FAK-I cotreatment with leptomycin B could be due to reduced ubiquitination, we immunoprecipitated CDK4/6 and immunoblotted for ubiquitin. FAK-I cotreatment with leptomycin B had no effect on FAK-I-mediated CDK4/6 ubiquitination (Supporting Data 5), suggesting that leptomycin B treatment may not trap a CDK4/6 deubiquitinase in the nucleus. These results are consistent with our previous observation that target protein degradation required transport to cytoplasmic proteasomal machinery (26, 27). In addition, immunostaining of B16F10 cells showed that cotreatment with MG-132 increased cytoplasmic accumulation of CDK4/6 (Supporting Data 6), further supporting that FAK-I promotes CDK4/6 ubiquitination in the nucleus (Supporting Data 5) and proteasomal degradation in the cytoplasm (Supporting Data 6).

To further verify the importance of nuclear FAK on CDK4/6 stability, we used adenovirus overexpressing either FERM-WT (predominantly localizing to the nucleus) or FERM-NLM (nonnuclear-localizing mutant; not localizing to the nucleus) (Supporting Data 4*D*) (31). While FERM-WT could reduce CDK4/6 in a dose-dependent manner, FAK-NLM had no effect on CDK4/6 expression (Fig. 2*E*). We also tested the effect of nuclear FAK on CDK4/6 stability by overexpressing either full-length FAK-WT (shuttling in and out of the nucleus) or FAK-KD (kinase dead; dominantly accumulating in the nucleus) (Supporting Data 4*C*) (30). While FAK-WT is primarily in the cytoplasm and had a modest effect on CDK4/6 expression, FAK-KD localizes to the nucleus and showed a larger decrease in CDK4/6 protein stability (Fig. 2*F*). Taken together, these data demonstrate that nuclear FAK promotes degradation of CDK4/6 through its FERM domain and that CDK4/6 are degraded in the cytoplasm following FAK inhibition.

To separate FAK cytoplasmic and nuclear signaling in reducing B16F10 growth, we used lentivirus to knockdown FAK in B16F10 cells. Furthermore, we overexpressed FLAG-FAK-WT or FLAG-FAK-NLM (Supporting Data 7*A*). Interestingly, shRNA knockdown of FAK in B16F10 cells increased CDK4 and CDK6 protein (Supporting Data 7*A*), potentially through less basal shuttling of FAK to the nucleus. Treatment with FAK-I was only able to reduce CDK4 expression in FLAG-FAK-WT B16F10 cells but not FLAG-FAK-NLM (Supporting Data 7*B*). However, FAK-I reduced proliferation of both FLAG-FAK-WT and FLAG-FAK-NLM B16F10 cells (Supporting Data 7*C*). FAK knockdown alone was enough to reduce B16F10 proliferation (Supporting Data 7*D*). Together, these data demonstrate that FAK expression and cytoplasmic signaling also contribute to B16F10 proliferation in addition to nuclear FAK–CDK4/6 axis.

### CDK4/6 stability is regulated by the APC/C CDH1 E3 ubiquitin ligase complex

**Figure 3:**
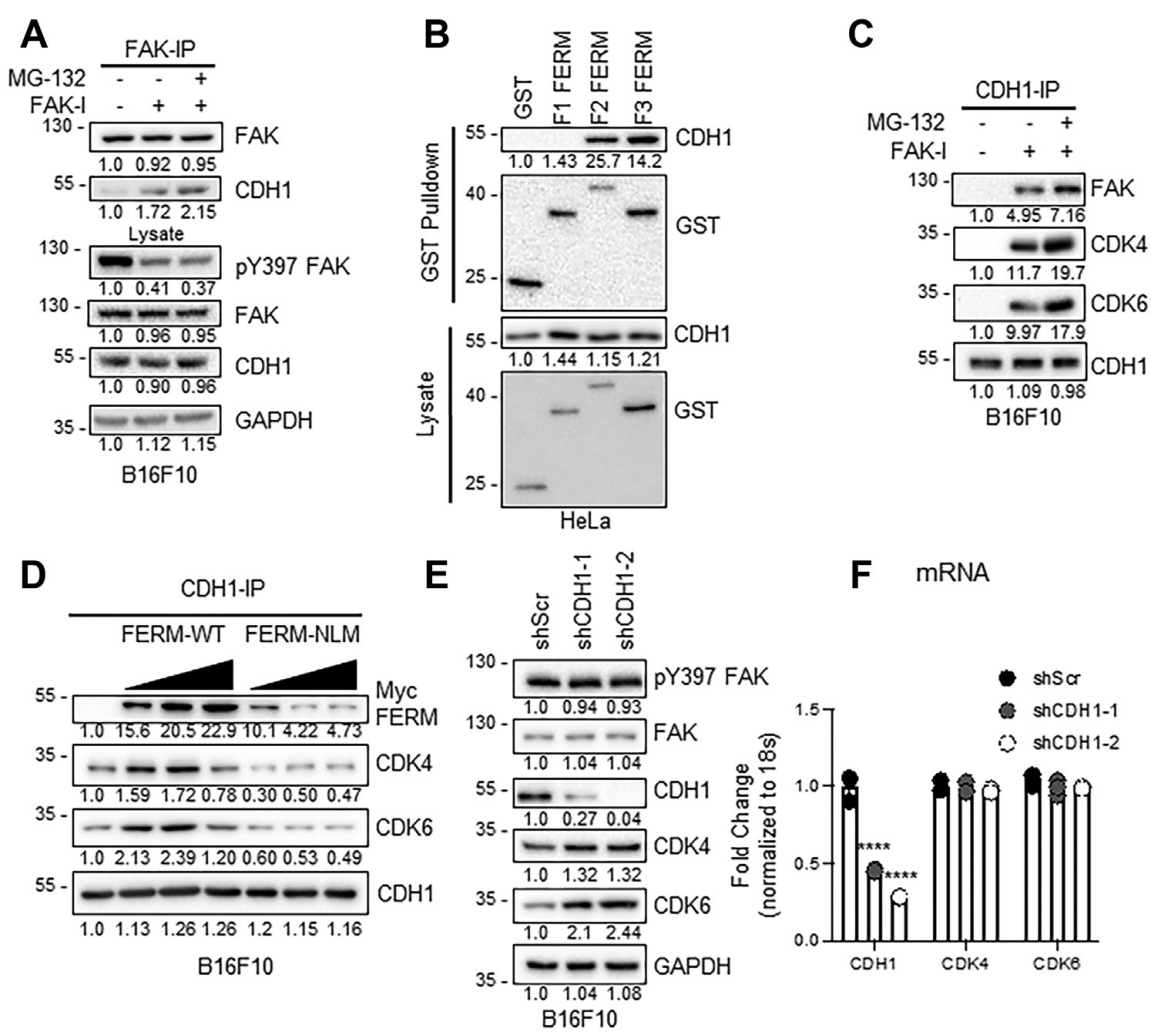
**FAK FERM recruitment of CDH1 is critical for CDK4/6 degradation.***A*, CDH1 associates with FAK following FAK inhibition. B16F10 cells were treated with FAK-I (PF-271, 2.5 μM) with or without MG-132 (10 μM) for 6 h. Immunoblotting of FAK-immunoprecipitates (IPs) for FAK and CDH1. Total lysates were probed for anti-pY397 FAK, FAK, CDH1, and GAPDH. Protein expression was normalized to either FAK (in IP) or GAPDH (in lysate), and fold change to untreated is shown (n = 3). *B*, CDH1 binds to the F2 and F3 FERM domains. HeLa cells were transfected with GST, GST-tagged F1, F2, or F3 FERM constructs. Immunoblotting of lysates and GST pulldown were blotted with CDH1 and GST (n = 3). Protein expression was normalized to either GST (in pulldown) or GAPDH (in lysate), and fold change to untreated is shown (n = 3). *C*, FAK inhibition increases association between CDH1 and CDK4/6. B16F10 cells were treated with FAK-I with or without MG-132 for 6 h. CDH1-IP were immunoblotted for CDH1, CDK4, and CDK6. Protein expression was normalized to CDH1 (IP), and fold change to untreated is shown (n = 3). *D*, FAK FERM-WT domain acts as a scaffold for CDH1–CDK4/6. B16F10 were transduced with increasing MOI of Myc-FERM-WT or Myc-FERM-NLM adenovirus. Immunoblotting of Myc-IP for CDH1, CDK4, CDK6, and Myc. Protein expression was normalized to CDH1 (in pulldown), and fold change to untreated is shown (n = 3). *E* and *F*, loss of CDH1 expression increases CDK4/6 protein expression. B16F10 cells were transduced with lentivirus expressing either scramble, CDH1-1, or CDH1-2 shRNA (shScr, shCDH1-1, shCDH1-2). *E*, immunoblotting was performed for pY397 FAK, FAK, CDK4, CDK6, CDH1, and GAPDH as loading control. Protein expression was normalized to GAPDH, and fold change to untreated is shown (n = 3). *F*, CDH1, CDK4, and CDK6 mRNA levels were determined *via* RT–qPCR (n = 3). CDH1, CDC homolog 1; CDK, cyclin-dependent kinase; FAK, focal adhesion kinase; FAK-I, FAK inhibitor; GST, glutathione-*S*-transferase; MOI, multiplicity of infection; pY397, autophosphorylation at tyrosine 397; qPCR, quantitative PCR.

To identify a potential E3 ligase responsible for FAK-mediated CDK4/6 degradation, we examined if FAK recruited the APC/C, an E3 ubiquitin ligase complex that regulates timely ubiquitination and degradation of various cell cycle mediators and has been implicated in promoting CDK4 degradation (33). During G1 phase, the APC/C complex requires activation by association with CDH1 (also known as FZR1), which is also responsible for substrate recruitment. Immunoblotting of FAK-IP showed increased association between CDH1 and FAK under FAK inhibition, which was further increased by cotreatment with MG-132 (Fig. 3*A*). As FAK FERM domain has been shown to act as a scaffold for substrates and their E3 ligases and we showed CDK4/6 binds the FERM domain (Fig. 2*B*) (27, 30, 31), we next evaluated which FERM lobe(s) associates with CDH1. GST pulldown of HeLa cells transfected with GST-FERM F1, F2, or F3 revealed that CDH1 preferentially associates with the F2 and F3 lobes (Fig. 3*B*). To further demonstrate a link between CDH1 and CDK4/6–FAK complex, we performed IP for endogenous CDH1 in B16F10 cells. Following FAK-I, CDK4/6 and FAK showed increased association with CDH1 that was further enhanced by cotreatment with MG-132 (Fig. 3*C*). To demonstrate a scaffolding role for FAK FERM domain in the recruitment of CDK4/6 and CDH1, we overexpressed increasing amounts of FERM WT and FERM NLM in B16F10 cells and performed CDH1-IP. As FERM WT expression increased, we saw reduced association between CDH1 and CDK4/6 (Fig. 3*D*), indicating that the FAK FERM domain does indeed act as a scaffold to recruit both CDH1 and CDK4/6. However, we did not observe any correlation in the association between CDH1 and CDK4/6 in FERM NLM–overexpressing cells (Fig. 3*D*). To further examine the scaffolding role of FAK FERM in CDK4/6–CDH1 recruitment, we examined a FAK FERM mutant (R312A/K313A) that has been shown to block recruitment of an E3 ligase but not its substrate (31). FERM R312A/K313A has been shown to localize to the nucleus but reduced ubiquitination of p53 by blocking recruitment of the E3 ligase mdm2 (Supporting Data 4*D*) (31). We overexpressed GFP-FERM WT, NLM, or R312A/K313A in B16F10 cells. Immunoblotting of GFP IPs revealed that CDH1 could associate with FERM WT but weakly bound to FERM R312A/K313A (Supporting Data 8*A*). However, CDK4/6 equally associated with FERM WT and R312A/K313A (Supporting Data 8*A*). Immunoblotting of CDK4/6 IPs revealed that indeed overexpression of FERM R312A/K313A failed to promote ubiquitination of CDK4/6, unlike FERM WT, which showed increased ubiquitination compared with vector control (Supporting Data 8*B*). These findings suggest that FAK FERM domain brings CDH1 and CDK4/6 together to promote ubiquitination and proteasomal degradation of CDK4/6.

To gain further insights into the role of CDH1 on CDK4/6 stability, we used shRNA to knockdown CDH1 in B16F10 cells. Two different CDH1 shRNAs (shCDH1-1 and shCDH1-2) significantly reduced CDH1 protein and mRNA expression (Fig. 3, *E* and *F*). Decreased CDH1 expression was associated with increased protein, but not mRNA expression of CDK4/6 (Fig. 3, *E* and *F*), suggesting that CDH1 is important for regulating CDK4/6 protein stability. To determine if FAK-mediated CDK4/6 degradation is dependent on CDH1, we treated B16F10 cells expressing shCDH1 with or without FAK-I. While FAK-I reduced CDK4/6 expression in shScr cells, FAK-I failed to reduce CDK4/6 levels in shCDH1-1 and shCDH1-2 B16F10 cells (Supporting Data 9). Together, these data demonstrate that nuclear FAK promotes CDK4/6 degradation through recruitment of the CDH1–APC/C E3 ubiquitin ligase complex.

### FAK inhibition reduces B16F10 melanoma tumor growth in mice

**Figure 4:**
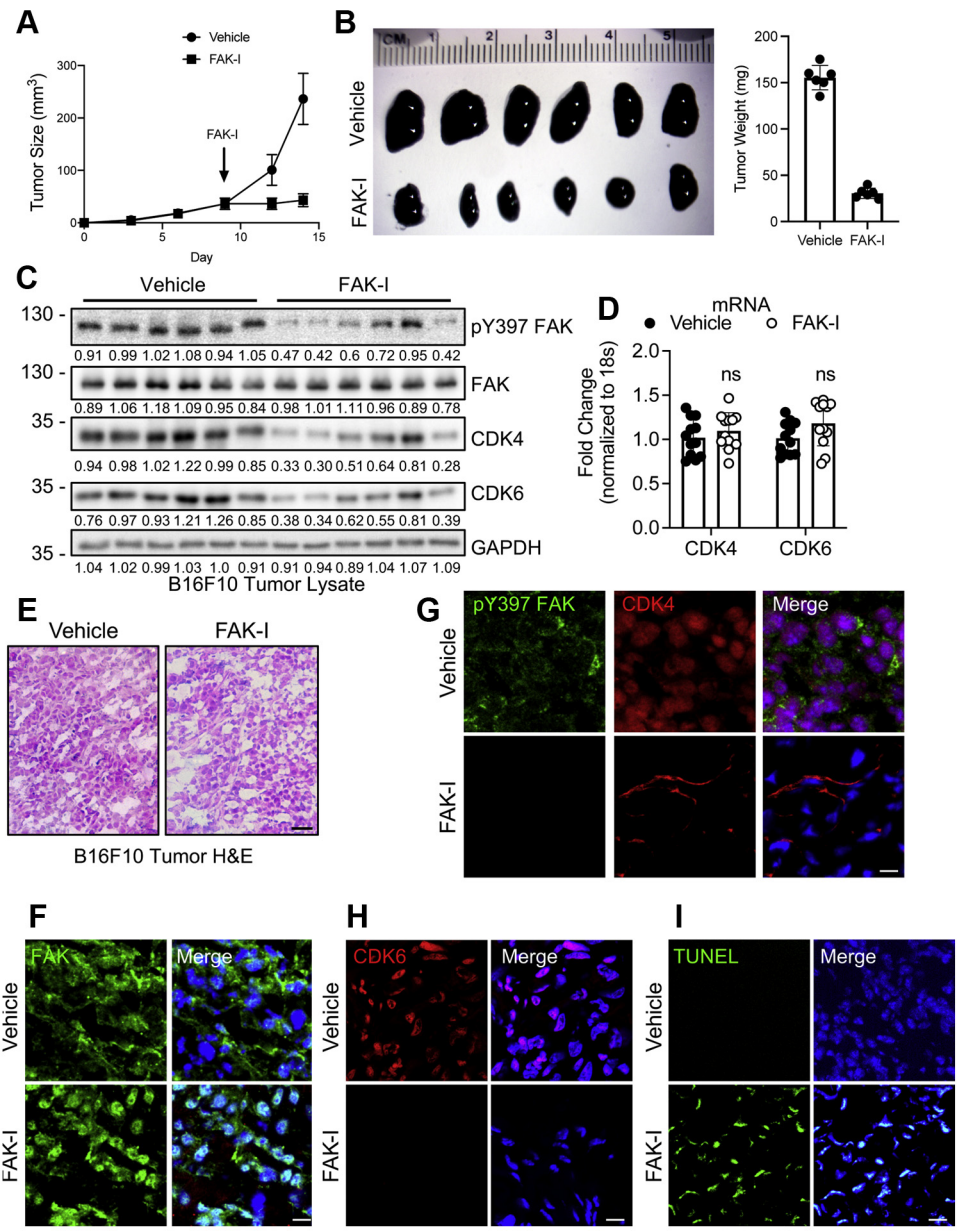
**FAK-I reduced CDK4/6 expression and tumor growth in orthotopic B16F10 flank mouse model.** Syngeneic B16F10 flank tumor model was performed in C57BL/6 mice. Starting on day 9, mice were treated with either vehicle or FAK-I (35 mg/kg) twice daily *via* oral gavage. *A*, tumor size was measured every 3 days for 14 days and plotted (n = 6, ±SD). *B*, at 14 days, tumors were collected and weighed (n = 6, ±SD). *C* and *D*, FAK inhibition reduced CDK4/6 protein expression, but not mRNA, in tumors. *C*, B16F10 tumor lysates were immunoblotted for pY397 FAK, CDK4, CDK6, and GAPDH as loading control; each lane indicates an individual tumor. Protein expression was normalized to GAPDH, and fold change to average densitometric intensity in vehicles is shown (n = 6). *D*, CDK4 and CDK6 mRNA levels were determined from B16F10 tumors *via* RT–qPCR (n = 6). *E*, representative H&E staining of vehicle and FAK-I-treated B16F10 tumors. The scale bar represents 200 μm. *F*, immunostaining of B16F10 tumors revealed that FAK inhibition increased FAK (*green*) nuclear localization within tumors. *Merge*: FAK (*green*) and DAPI (*blue*). The scale bar represents 50 μm. *G*, immunostaining of B16F10 tumors showed that reduced active pY397 FAK (*green*) was associated with loss of CDK4 (*red*) within tumors. *Merge*: pY397 FAK (*green*), CDK4 (*red*), and nuclei (*blue*, DAPI). The scale bar represents 50 μm. *H*, immunostaining of B16F10 tumors showed that FAK-I-treated tumors had decreased CDK6 (*red*) staining compared with vehicle-treated tumors. *Merge*: CDK6 (*red*) and nuclei (*blue*, DAPI). The scale bar represents 50 μm. *I*, FAK inhibition increased apoptosis of tumor cells. Apoptosis in B16F10 tumors was analyzed by TUNEL assay (*green*). *Merge*: TUNEL assay (*green*) and nuclei (*blue*, DAPI). The scale bar represents 50 μm. CDK, cyclin-dependent kinase; DAPI, 4′,6-diamidino-2-phenylindole; FAK, focal adhesion kinase; FAK-I, FAK inhibitor; pY397, autophosphorylation at tyrosine 397; qPCR, quantitative PCR.

To evaluate if FAK inhibition could reduce tumor cell proliferation *in vivo*, we injected B16F10 cells into the flank of C57BL/6 mice. On day 9, mice were treated with either vehicle or FAK-I twice daily by oral gavage for 5 days. Mice treated with FAK-I displayed reduced tumor growth compared with vehicle-treated mice (Fig. 4*A*). In addition, final tumor size and weight were significantly decreased in FAK-I-treated mice compared with vehicle (Fig. 4, *A* and *B*). To determine if FAK inhibition reduced CDK4/6 expression *in vivo*, we analyzed tumor lysates *via* immunoblotting. Tumors collected from FAK-I-treated mice show decreased active pY397 FAK (Fig. 4*C*), demonstrating drug efficacy. FAK-I-treated tumors also show decreased CDK4/6 protein, but not mRNA expression (Fig. 4, *C* and *D*), suggesting that FAK inhibition reduced CDK4/6 protein stability *in vivo*.

We next performed immunohistochemical staining of B16F10 tumors to further examine the effect FAK inhibition had on FAK and CDK4/6 *in vivo*. From H&E staining, FAK-I group exhibited less dense tumor area compared with vehicle (Fig. 4*E*). While vehicle-treated tumors showed primarily cytoplasmic FAK localization, treatment with FAK-I increased FAK nuclear localization within tumors (Fig. 4*F*). Furthermore, high levels of active pY397 FAK were observed in vehicle-treated tumors compared with the FAK-I group (Fig. 4*G*). Increased FAK nuclear localization in tumors was associated with decreased CDK4 and CDK6 expression (Fig. 4*H*). Mice treated with FAK-I also showed reduced cyclin D1 expression compared with vehicle-treated mice (Supporting Data 10). Finally, we evaluated apoptosis in B16F10 tumors using TUNEL assay. While vehicle-treated tumors showed little signal, FAK-I tumors showed significant TUNEL signal within nuclei (Fig. 4*I*). Together, these data show that FAK inhibition increases FAK nuclear localization leading to loss of CDK4/6 within tumor cells and leading to increased tumor cell apoptosis *in vivo*.

**Figure 5:**
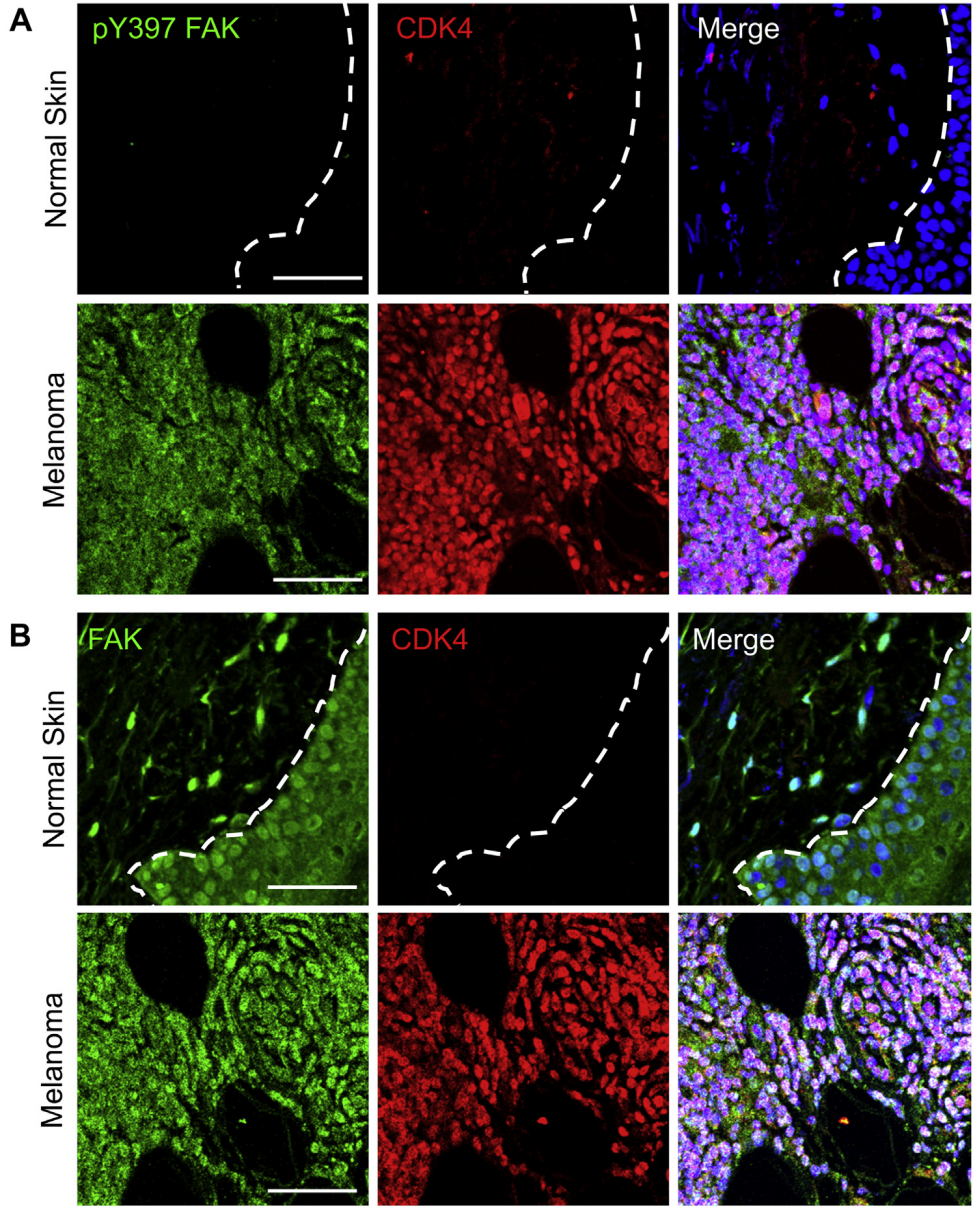
**Increased active cytoplasmic FAK and CDK4 expression in human melanoma.** Human melanoma lesions and patient-matched normal skin were embedded in paraffin and sectioned for immunostaining. *A*, normal skin showed low levels of active pY397 FAK (*green*) and CDK4 (*red*) compared with melanoma lesions. *Merge*: pY397 FAK (*green*), CDK4 (*red*), and nuclei (*blue*, DAPI). *B*, melanoma lesions showed increased cytoplasmic FAK localization (*green*) compared with mostly nuclear staining within normal skin. *Merge*: FAK (*green*), CDK4 (*red*), and nuclei (*blue*, DAPI). *Dashed line*: Epidermis and dermis boundary. The scale bar represents 50 μm. CDK, cyclin-dependent kinase; DAPI, 4′,6-diamidino-2-phenylindole; FAK, focal adhesion kinase; pY397, autophosphorylation at tyrosine 397.

We next looked at changes in FAK activation and localization within human melanoma specimen. Immunostaining revealed that FAK was mostly inactive and localized to the nuclei of cells within patient-matched normal skin (Fig. 5 and Supporting Data 11 and 12). Increased nuclear FAK localization was correlated with low levels of CDK4 expression (Fig. 5*B*, Supporting Data 11 and 12). However, FAK showed increased activity and cytoplasmic localization within the melanoma lesion (Fig. 5, Supporting Data 11 and 12). CDK4 expression was also elevated within the melanoma lesion (Fig. 5, Supporting Data 11 and 12). While these findings are preliminary because of the small sample size (n = 2), these data support the notion that FAK activity and cytoplasmic localization are increased within melanoma, thus enhancing nuclear FAK by FAK-Is would repress CDK4 expression and melanoma progression.

## Discussion

**Figure 6:**
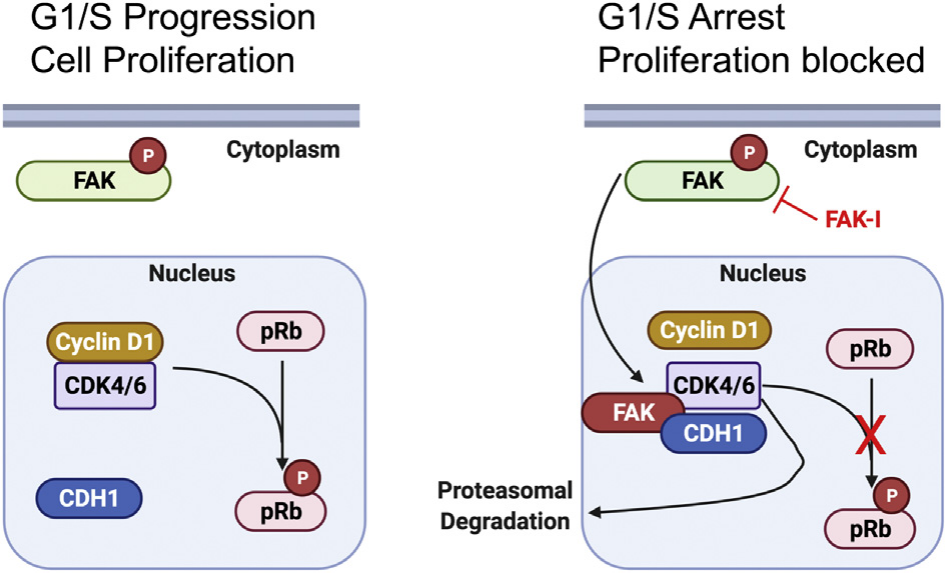
**Nuclear FAK regulation of CDK4/6 in cell cycle progression.** Forced FAK nuclear localization promotes G1/S arrest by disrupting CDK4/6–cyclin D1 complex and promoting CDH1-mediated CDK4/6 proteasomal degradation. *Left*, within proliferating tumor cells, FAK is active and primarily localized to the cytoplasm. *Right*, FAK inhibition forces FAK to the nucleus and promoting CDK4/6 degradation *via* CDH1. Loss of CDK4/6 reduces phosphorylation of pRb, thus leading to G1/S-cell cycle arrest. CDH1, CDC homolog 1; CDK, cyclin-dependent kinase; FAK, focal adhesion kinase; pRb, retinoblastoma protein.

In the present study, we demonstrated that FAK inhibition increased FAK nuclear localization in B16F10 melanoma cells, leading to decreased stability of CDK4/6 protein and cell cycle arrest. Mechanistically, the FAK FERM domain acted as a scaffold to promote CDK4/6 degradation through recruitment of CDH1, part of the E3 ubiquitin ligase APC/C (Fig. 6). The importance of nuclear FAK in CDK4/6 regulation was further demonstrated by failure of FERM NLM to reduce CDK4/6 protein. Interestingly, FAK inhibition seemed to only affect the stability of CDK4/6 but not CDK1/2. The cell cycle–related CDKs are divided into three groups based on their homology and the cyclins they interact with: (1) CDK1/2/3, (2) CDK4/6, and (3), CDK5/14/15/16/17/18 (34). Of these three groups, CDK4/6 has no yeast ortholog and are only expressed within eumetazoans. Analysis of crystal structures demonstrated that CDK2 interacts with cyclin A at both their N-terminal and C-terminal lobes, resulting in a conformation change activating CDK2 (34). However, CDK4 and D-type cyclins only interact with their N-terminal lobe and does not induce an active conformation within CDK4 (34). These small differences in evolutionary origin and interactions with their cyclins could explain why FAK can associate with CDK4/6 but not CDK1/2. Examination of other CDKs and their ability to bind FAK will need to be explored.

Interestingly, we observed that FAK inhibition also decreased D-type cyclin protein expression after only 6 h (Fig. 1*D* and Supporting Data 3*A*). While FAK-I had no effect on D-type cyclin mRNA at 6 h (Supporting Data 3*B*), 24, and 48 h, FAK-I treatment reduced D-type cyclin mRNA (Supporting Data 3, *C*–*E*). It is possible that FAK reduced D-type cyclin transcription as we previously demonstrated that FAK-I could reduce cyclin D1 transcription in smooth muscle cells through FAK-mediated degradation of GATA4 (26). However, loss of D-type cyclin protein preceded decreased mRNA expression, suggesting that FAK inhibition may have reduced D-type cyclin stability as well. It has been shown that mutant cyclin D1 (K114E), which fails to bind and activate CDK4, is ubiquitinated and degraded (32). FAK-mediated loss of CDK4 coupled with the short half-lives (<30 min) of D-type cyclins could be why we see loss of D-type cyclin protein expression prior to changes in mRNA (35, 36, 37). However, more studies need to be performed looking at CDK4-mediated stability of D-type cyclins.

While BRAF inhibitors have been approved to treat melanoma in patients with activating BRAF mutations, several patients have innate or acquired resistance to BRAF inhibitors (38). Analysis of patient tumors prior to treatment with a BRAF inhibitor demonstrated that patients with increased cyclin D1 copy number and low p16INK4A expression were shown to have reduced progression-free survival (39), suggesting that hyperactivation of CDK4/6-p16INK4A pathway may promote resistance to BRAF inhibitors. This was further demonstrated in a study that showed overexpressing cyclin D1 in a melanoma cell line could promote BRAF inhibitor resistance (39). Analysis of melanoma patients who developed BRAF inhibitor resistance revealed increased mitogen-activated protein kinase signaling from either development of NRAS and KRAS mutations in progressive tumors, or through 2- to 15-fold increase in BRAF mRNA compared with patient-matched baseline tumors (40). This study also showed increased activation of the PI3K–phosphatase and tensin homolog–AKT pathway, suggesting that several pathways play a role in BRAF inhibitor resistance. In BRAF mutant colorectal cancer cells, BRAF inhibitor treatment increased FAK activation leading to increased Wnt pathway signaling (24). However, whether BRAF inhibitor resistant melanoma shows a similar pattern of increased FAK activity remains to be examined.

While CDK4/6 inhibitors have been approved for use in certain cancers, there are still patients who show no response to this therapy or develop resistance over time. One study demonstrated that MET/FAK signaling could bypass CDK4/6-dependent activation of CDK2 through upregulation of SKP2, a ubiquitin ligase subunit that regulates stability of cell cycle inhibitor p21 (25). However, FAK inhibition could overcome CDK4/6 inhibitor resistance by increasing p21 expression (25). While they demonstrated the potential effectiveness of combinatorial CDK4/6 and FAK-I treatment *in vitro*, our present study suggests that FAK-Is could be used as an alternative treatment for CDK4/6 inhibitor resistant tumors.

Apart from its role in cell cycle progression, a recent study demonstrated that CDK4 regulated expression of PD-L1, which made tumors more susceptible to anti-PD-L1 therapy (41). This study found that CDK4 inhibition increased PD-L1 protein stability by inhibiting CDH1 and blocking cullin 3–Speckle Type POZ Protein (SPOP)–mediated degradation of PD-L1 (41). Elevated expression of PD-L1 on the surface of tumor cells allows them to evade the immune system by binding to PD-1, which inhibits T-cell activity. While we observed that FAK inhibition could reduce B16F10 tumor growth, it still remains to be seen if FAK-I-mediated loss of CDK4/6 protein increases PD-L1 expression in B16F10 cells and could eventually lead to escape from the immune system. However, in tumors that show resistance to anti-PD-L1 or anti-PD-1 therapy, forced expression of PD-L1 could sensitize them to these antibody therapies (41).

Despite numerous studies demonstrating increased FAK expression and activation in numerous cancers and the potential efficacy in FAK-Is to treat cancer (23), there is little known regarding FAK inhibition in melanoma. Increased FAK activation has been observed in aggressive melanoma and that overexpression of the endogenous FAK-I FRNK could reduce melanoma cell migration and invasion (42, 43). Examining our human melanoma specimens with patient-matched normal tissue, we found that FAK was primarily inactive and nuclear localized in normal skin, which was associated with low levels of CDK4 (Fig. 5). However, melanoma lesions showed elevated cytoplasmic-active FAK and increased CDK4 expression (Fig. 5). Furthermore, another study found that increased FAK activity in uveal melanoma was required for YAP (Yes1 associated transcriptional regulator)-mediated aberrant cancer cell growth and that FAK inhibition could reduce uveal melanoma growth (44). However, the genetic alterations that give rise to uveal melanoma (*i.e.*, *GNAQ*) differ from those in cutaneous melanoma (*i.e.*, *BRAF*, *CDKN2A*, *CDK4*), suggesting that different pathways may be involved in their progression. While our study here provides evidence for FAK inhibition in reducing melanoma progression, in part, through decreased CDK4/6 expression and cell cycle arrest, more work is needed to evaluate the potential of FAK-Is in treating melanoma either as a monotherapy or in combination with BRAF, MEK, or immune checkpoint therapies.

Interestingly, it appears that FAK not only regulates cell cycle progressors such as CDK4/6 and cyclin D (26) but also regulates expression of cell cycle inhibitor proteins such as p21 and p27 (27). Furthermore, FAK nuclear localization appears to be important for regulating both arms of the cell cycle in multiple cell types. As such, in addition to the inhibitory effect of FAK-I in the cytoplasmic FAK signaling, FAK-Is could prove beneficial in reducing tumor cell proliferation through forced FAK nuclear localization and shifting the balance of cell cycle regulators toward the inhibitory arm (*i.e.*, increased p21/p27 and reduced cyclin D-CDK/46).

## Experimental procedures

### Antibodies and reagents

FAK (catalog no.: 05-537; mouse), PCNA (catalog no.: PLA0080; rabbit), GST (catalog no.: 05-782), ubiquitin (catalog no.: ST1200), and GAPDH (catalog no.: MAB374; mouse) antibodies were purchased from Millipore; pY397 FAK (catalog no.: 44-624G; rabbit) and cyclin D1 (catalog no.: MA5-16356; rabbit) antibodies were purchased from Life Technologies; CDK4 (catalog no.: sc-166373; mouse), CDK6 (catalog no.: sc-7961; mouse), CDK1 (catalog no.: sc-54; mouse), CDK2 (catalog no.: sc-6248), CDH1 (catalog no.: sc-56312; mouse), Myc (catalog no.: sc-40; mouse), and cyclin D3 (catalog no.: sc-6283; mouse) antibodies were purchased from Santa Cruz Biotechnology; p-pRb (catalog no.: 8516; rabbit) antibody was purchased from Cell Signal Technology; cyclin D2 (catalog no.: 554201; mouse) antibody was purchased from BD Biosciences; GFP (catalog no.: MMS-118P) antibody was purchased from Covance; FAK-I PF-271 was purchased from MedKoo; MG-132 was purchased from Selleckchem; cycloheximide and FLAG (catalog no.: F3165; mouse) antibody were purchased from Sigma; and leptomycin B was purchased from LC Laboratories.

### Human melanoma specimen

Patient-matched human healthy and melanoma specimens were obtained from the University of South Alabama Health Biobank as paraffin blocks. Specimens were sectioned on a microtome at 5 μM and deparaffinized prior to immunostaining.

### Cells

B16F10, HeLa, A375, and RPMI7951 cells were purchased from American Type Culture Collection. WM115 and WM266-4 cells were purchased from Rockland Immunochemicals. HEK 293FT cells were purchased from Life Technologies. Cells were cultured in Dulbecco's modified Eagle's medium containing 10% fetal bovine serum. HeLa cells were transfected with GFP, GFP-FAK, GFP-FERM, GFP-kinase, or GFP-FRNK constructs using polyethylenimine (Polysciences) (31). B16F10 cells were transduced with adenovirus encoding for either FAK-WT, FAK-KD, FAK FERM-WT, or FAK FERM-NLM.

### Proliferation assay

About 1 × 10^4^ cells were plated into a 6-well plate. Every 24 h, cells were collected *via* trypsinization and enumerated using Countess II automatic cell counter (Invitrogen). Starting at 24 h, cells were treated with either vehicle or PF-271 (2.5 μM). Trypan blue (Bio-Rad)–positive cells were excluded from live cell counts.

### Cell cycle analysis and TUNEL staining

B16F10 melanoma cells were treated with either vehicle or PF-271 (2.5 μM) for either 24 or 48 h. Cells were collected *via* trypsinization, and DNA was stained using propidium iodide. Cells were analyzed *via* flow-assisted cell sorting on BD Biosciences CANTO II. The percentage of cells in each phase of the cell cycle was determined. TUNEL apoptosis detection kit (4812-30-K) was purchased from R&D Systems and performed following the manufacturer’s instructions.

### Immunoblotting

Cells were lysed in Triton lysis buffer (1% Triton X-100, 50 mM Hepes [pH 7.4], 150 mM NaCl, 10% glycerol, 1.5 mM MgCl_2_, 1 mM EGTA, 10 mM sodium pyrophosphate, 100 mM NaF, 1 mM NaVO_4_, 1× Protease Inhibitor Cocktail [Roche]). Following sonication and centrifugation, the supernatant was mixed with loading buffer, and samples were separated *via* SDS-PAGE. Proteins were transferred to polyvinylidene difluoride membranes and incubated with primary antibodies overnight at 4 °C with rocking. Immunoblot densitometry was determined using ImageJ (National Institutes of Health). Fold change over control group following normalization to GAPDH is indicated below each blot (n = 3).

### Lentiviral production

Lentivirus was produced using a third-generation packaging system in HEK 293FT cells. Cells were transfected using polyethylenimine reagent; after 72 h, lentivirus-containing medium was centrifuged to remove cell debris and passed through a 0.45 mm filter (Steriflip-HV polyvinylidene difluoride; Millipore). Lentivirus was concentrated using Amicon Ultra-15 Centrifuge Filter (100,000 Da cutoff; Millipore) (26). Lentivirus was aliquoted and stored at −80 °C until used. B16F10 cells were transduced with lentivirus encoding FLAG-FAK-WT, FLAG-FAK-KD, FLAG-FAK-NLM, or shRNAs for scramble control (shScr), CDH1 (shCdh1-1and shCdh1-2), and FAK. Primers used for shRNA: shCDH1-1, Forward: 5′-TGC ACG CCA ATG AGC TGG TGT TCA AGA GAC ACC AGC TCA TTG GCG TGC TTT TTT C-3′ and Reverse: 5′-TCG AGA AAA AAG CAC GCC AAT GAG CTG GTG TCT CTT GAA CAC CAG CTC ATT GGC GTG CA-3′; shCDH1-2, Forward 5′-TGG ACG CCA CCT CGG ACA ATT TCA AGA GAA TTG TCC GAG GTG GCG TCC TTT TTT C-3′ and Reverse: 5′-TCG AGA AAA AAG GAC GCC ACC TCG GAC AAT TCT CTT GAA ATT GTC CGA GGT GGC GTC CA-3′; shFAK: Forward 5′-TGA AGG GAT CAG TTA CCT GAT TCA AGA GAT CAG GTA ACT GAT CCC TTC TTT TTT C-3′ and Reverse: 5′-TCG AGA AAA AAG AAG GGA TCA GTT ACC TGA TCT CTT GAA TCA GGT AAC TGA TCC CTT CA-3′. After 3 days, cells were selected with puromycin (2 mg/ml) for 2 days. CDH1 knockdown was verified by RT–quantitative PCR (qPCR) and immunoblotting.

### RT–qPCR

Total mRNA was isolated from B16F10 cells treated with FAK-I or stably expressing shScr, shCdh1-1, or shCdh1-2 using NucleoSpin RNA isolation kit (Macherey–Nagel). Complementary DNA was synthesized using SuperScript III Reverse Transcriptase (Life Technologies). RT–qPCR was performed (CFX Connect and iTaq Universal SYBR Green SMX; Bio-Rad). All PCRs were performed with the following steps: initial denaturation, 95 °C for 10 min; 40 cycles of denaturation, 95 °C for 15 s, and annealing/extension, 60 °C for 60 s. Primers used: mouse Cdh1, Forward: 5′-TGC CCT GTG TTT CAG AGA TG-3′ and Reverse: 5′-GGA AGT TCA CGC TCC AGT T-3′; mouse CDK4, Forward: 5′-TCT ACA GCT ACC AGA TGG-3′ and Reverse: 5′-AAC TGG TCG GCT TCA GAG-3′; mouse CDK6, Forward: 5′-GTG TCG GTT GCA TCT TTG-3′ and Reverse: 5′-CCT AGG CCA GTC TTC CTC-3′; mouse cyclin D1, Forward: 5′-TGG TGA ACA AGC TCA AGT GG-3′ and Reverse: 5′-GCA GGA GAG GAA GTT GTT GG-3′; mouse cyclin D2, Forward: 5′-CGA AGG ATG TGC TCA ATG AA-3′ and Reverse: 5′-TTA CCT GGA CCG TTT CTT GG-3′; mouse cyclin D3, Forward: 5′-ACG CCC CTG ACT ATT GAG AA-3′ and Reverse: 5′-ACA GAG GGC CAA AAA GGT CT-3′; and mouse 18s, Forward: 5′-CTT AGA GGG ACA AGT GGC G-3′ and Reverse: 5′-ACG CTG AGC CAG TCA GTG TA-3′.

### Immunoprecipitation

B16F10 cells were plated into plastic tissue culture dishes and after 3 h treated with PF-271 (2.5 μM) with or without proteasome inhibitor (MG-132, 10 μM) for 6 h. Cells were lysed with immunoprecipitation buffer (1% Triton X-100, 50 mM Hepes, 150 mM NaCl, 10% glycerol, 1 mM EDTA, 10 mM sodium pyrophosphate, 100 mM NaF, 1 mM NaVO_4_, and cOmplete protease inhibitor cocktail [Roche]). Lysates were cleared by centrifugation, and equal amounts of proteins were subjected to immunoprecipitation with indicated antibodies. The lysates were rotated overnight at 4 °C, then protein G or A agarose beads were added, and the mixture was rotated for 2 h at 4 °C. The immunocomplexes were washed three times with immunoprecipitation buffer and suspended with 2× SDS-loading buffer. Samples were separated by SDS-PAGE and immunoblotted with indicated antibodies.

### GST pull-down assay

GST-FERM F1, F2, and F3 subdomain constructs were transfected in HeLa cells (31). GST-FERM subdomains were pulled down using glutathione beads to determine their interaction with endogenous CDK4, CDK6, and CDH1.

### Transfection of GFP-FERM mutants

B16F10 cells were transfected with either GFP-FERM-WT, GFP-FERM-NLM, or GFP-FERM-R312A/K313A constructs using Lipofectamine 2000 (Life Technologies) for 24 h. Cells were then subjected to either GFP immunoprecipitation or fluorescent imaging.

### B16F10 syngeneic tumor model

All animal experiments were approved by the Institutional Animal Care and Use Committee at the University of South Alabama. About 1 × 10^6^ B16F10 melanoma cells were injected into the flank of male and female C57BL/6 mice (n = 6). Tumor size was measured every 3 days for the duration of the experiment. Starting on day 9, mice were treated with either vehicle (30% [2-hydroxypropyl]-β-cyclodextrin/3% dextrose) or PF-271 (35 mg/kg) twice daily *via* oral gavage. On day 14, mice were euthanized, and tumors were collected, imaged, and weighed. Each tumor was cut into pieces for either immunoblotting, RNA isolation, or preserved in optimal cutting temperature compound for frozen sectioning.

### Immunostaining

B16F10 cells on fibronectin-coated coverslips or frozen tumor sections were fixed with paraformaldehyde and permeabilized with 0.3% Triton X-100. Frozen tissue sections were fixed with cold acetone for 15 min. Samples were blocked (1% bovine serum albumin and 1% goat serum) for 1 h at room temperature and incubated with primary antibody overnight at 4 °C. Samples were incubated with secondary antibody (1:1000 dilution) for 1 h at room temperature. Species-specific immunoglobulin G or secondary antibodies were used as negative control. Nuclei were stained with 4′,6-diamidino-2-phenylindole (Sigma). Slides were mounted (Fluoromount-G; SouthernBiotech), and images were acquired with a confocal microscope (Nikon A1R; Nikon). Images were processed (Photoshop CS5; Adobe).

### Statistical analysis

Datasets underwent Shapiro–Wilk test for normality, and statistical significance between experimental groups was determined with Student's *t* test or one-way ANOVA with Sidak multiple comparisons test (GraphPad Prism; GraphPad Software, Inc). Power analyses were performed to determine sample size for one-way ANOVA.

## Data availability

All data are contained within the article.

## Supporting information

This article contains supporting information.

## Conflict of interest

The authors declare that they have no conflicts of interest with the contents of this article.
