# Supplementary material for: Nuclear focal adhesion kinase induces APC/C activator protein CDH1-mediated cyclin-dependent kinase 4/6 degradation and inhibits melanoma proliferation
Source: J Biol Chem. 2022 May 5;298(6):102013. doi: 10.1016/j.jbc.2022.102013 (PMC9163754; doi:10.1016/j.jbc.2022.102013)
Supplement: Supporting Data 1–12 Legends [file mmc1.docx]

**Supporting Data Legends**

**Nuclear focal adhesion kinase induces APC/C activator protein CDH1-mediated cyclin-dependent kinase4/6 degradation and inhibits melanoma proliferation**

James M. Murphy^1^, Kyuho Jeong^2^, Eun-Young Erin Ahn^3^, and Ssang-Taek Steve Lim^1*^

1. Department of Pathology, University of Alabama at Birmingham, Birmingham, AL 35294

2. Department of Biochemistry and Molecular Biology, College of Medicine, University of South Alabama, Mobile, AL 36688

3. Department of Pathology, O'Neal Comprehensive Cancer Center, University of Alabama at

Birmingham, Birmingham, AL 35294

**Running title**: Nuclear FAK control of CDH1-mediated CDK4/6 turnover

*Correspondence to Ssang-Taek Steve Lim, Ph.D., Department of Pathology, University of Alabama at Birmingham, 1825 University Blvd, Shelby 815,

Birmingham, AL 35294. Phone: (205) 996-4659. Email: [stlim@uab.edu](mailto:stlim@uab.edu)

**Key words:** FAK, CDK4/6, CDH1, melanoma

**Supporting Data Legends**

**Supporting Data 1. FAK inhibition reduces CDK4/6 protein in several human melanoma cell lines.** The human melanoma cell lines **(A)** A375, **(B)** RPMI 7951, and **(C)** WM115 cells were treated with FAK-I (2.5 μM) with or without MG-132 (10 μM) for 6 h. Immunoblots for pY397 FAK, CDK4, CDK6, and GAPDH as loading control. Protein expression was normalized to GAPDH and fold change over untreated is shown (n=3).

**Supporting Data 2. FAK inhibition reduces proliferation of human melanoma cell lines.** The human melanoma cell lines **(A)** WM115, **(B)** WM266-4, **(C)** A375, and **(D)** RPMI 7951 were treated with FAK-I (2.5 ) at 24h, and cells were enumerated every 24 h for the indicated duration. *****P*<0.0001. **(E)** Immunoblots of human melanoma cell lines after 48 h FAK-I treatment for active pY397 FAK , FAK, and GAPDH as loading control (n=3).

**Supporting Data 3. FAK inhibition reduces D-type cyclin expression.** B16F10 cells were treated with FAK-I (2.5 μM) for indicated times. **(A)** Immunoblots for cyclin D1, cyclin D2, cyclin D3, GATA4, p-pRb, pRb, and GAPDH as loading control. Protein expression was normalized to GAPDH and fold change over untreated is shown (n=3). **(B-F)** mRNA expression was evaluated via RT-qPCR (n=3). ****P*<0.001*, ****P<*0.0001 **(G)**  Cyclin D1 was overexpressed in HEK 293T cells and immunoblotting of cyclin D1 immunoprecipitates (IP) for ubiquitin was performed.

**Supporting Data 4. Schematic and localization of FAK or FERM constructs.** Amino acid ranges for **(A)** GFP-FAK, FERM, Kinase, and FRNK constructs; or **(B)** GST-FERM F1, F2, F3 constructs are illustrated. **(C)** Immunostaining for FLAG-FAK-WT, FLAG-FAK-KD (kinase dead), and FLAG-FAK-NLM (green) overexpressed in B16F10 cells. Merge: FLAG-FAK mutants (green) and nuclei (blue, DAPI). **(D)** Images of GFP-FERM-WT, GFP-FERM-NLM, and GFP-FERM-R312A/K313A (green) overexpression in B16F10 cells. Merge: GFP-FAK mutants (green) and nuclei (blue, DAPI). Scale bars: 20 μm.

**Supporting Data 5. Leptomycin B does not reduce FAK-I mediated CDK4/6 ubiquitination.** B16F10 cells were treated with FAK-I (2.5 μM) with or without Leptomycin B (20 ng/ml) for 6 h. Immunoblots for ubiquitin was performed from on either **(A)** CDK4-IP or **(B)** CDK6-IP.

**Supporting Data 6. FAK promotes CDK4/6 degradation in the cytoplasm.** B16F10 cells were treated with FAK-I (2.5 μM) with or without either Leptomycin B (20 ng/ml) or MG-132 (20 μM) for 6 h. FAK-I-induced CDK4/6 degradation occurs in the cytoplasm. Immunostaining for FAK, pY397 FAK, CDK4, and CDK6 are shown (n=3). Images of Vehicle, FAK-I, and FAK-I + Leptomycin B are from Figure 2D to compare with FAK-I + MG132 staining. Scale bar: 20 μm.

**Supporting Data 7. FAK-I still reduces B16F10 growth in FAK-WT and FAK-NLM expressing cells.** B16F10 cells were transduced with lentivirus expressing shRNA against FAK and transduced to express either FLAG-FAK-WT, FLAG-FAK-KD, or FLAG-FAK-NLM. **(A)** Immunoblots of total FAK, FLAG-FAK mutants, CDK4, CDK6, and actin as loading control. **(B)** B16F10 shFAK cells expressing either FLAG-FAK-WT or FLAG-FAG-NLM were treated with FAK-I (2.5 μM) for 6 h. Immunoblots for pY397 FAK, FLAG-FAK mutant, CDK4, and actin as loading control. **(C)** Proliferation assay of B16F10 shFAK cells expressing either FLAG-FAK-WT or FLAG-FAG-NLM were performed with or without FAK-I (2.5 μM). Cells were enumerated every 24 h. *****P*<0.0001 (FAK-WT Vehicle vs FAK-WT FAK-I; FAK-NLM Vehicle vs FAK-NLM FAK-I). **(D)** Proliferation assay of shScr and shFAK B16F10 cells. Cells were enumerated every 24 h. *****P*<0.0001.

**Supporting Data 8. FERM R312A/K313A mutant fails to promote CDK4 ubiquitination by failing to bind CDH1.** B16F10 cells were transfected with GFP-FERM WT, NLM, or R312A/K313A mutants. **(A)** Immunoblotting of GFP immunoprecipitates for CDH1, CDK4, and GFP-FERM. FERM-NLM failed to bind both CDK4 and CDH1, but FERM-R312A/K313A weakly bound CDH1. **(B)** FERM-WT but not FERM-NLM or FERM-R312A/K313A increased CDK4 ubiquitination.

**Supporting Data 9. FAK requires CDH1 to promote CDK4 degradation.** B16F10 cells were transduced with lentivirus expressing either scramble, CDH1-1, or CDH1-2 shRNA (shScr, shCDH1-1, or shCDH1-2). FAK inhibition failed to reduce CDK4 protein in CDH1-1 and CHD1-2 shRNA B16F10 cells.

**Supporting Data 10. FAK inhibition reduces cyclin D1 expression in B16F10 tumors.** Immunostaining of vehicle and FAK-I treated B16F10 tumors for FAK (green) and cyclin D1 (red). Merge: FAK (green), cyclin D1 (red), and nuclei (blue, DAPI). FAK staining from Figure 4F was shown to compare with cyclin D1 expression and localization. Scale Bar: 100 μm.

**Supporting Data 11. Identification of various structures within normal skin.** Immunostaining of normal skin for FAK (green), CDK4 (red), and nuclei (blue, DAPI). Sebaceous glands, sweat glands, and superficial vascular plexus are indicated. Superficial vascular plexus is shown in Figure 5. Scale bar: 500 μm.

**Supporting Data 12. Immunostaining of human melanoma for FAK, CDK4, and active pY397 FAK.** Immunostaining of human melanoma. **(A)** FAK (green) showed cytoplasmic localization associated with high levels of CDK4 (red). Merge: FAK (green), CDK4 (red) and nuclei (blue, DAPI). **(B)** Elevated pY397 FAK (green) levels associated with CDK4 (red) expression. Merge: pY397 FAK (green), CDK6 (red), and nuclei (blue, DAPI). Scale bars: 50 μm.
