# Supplementary figures and images for: Nuclear focal adhesion kinase induces APC/C activator protein CDH1-mediated cyclin-dependent kinase 4/6 degradation and inhibits melanoma proliferation
Source: J Biol Chem. 2022 May 5;298(6):102013. doi: 10.1016/j.jbc.2022.102013 (PMC9163754; doi:10.1016/j.jbc.2022.102013)

**A**

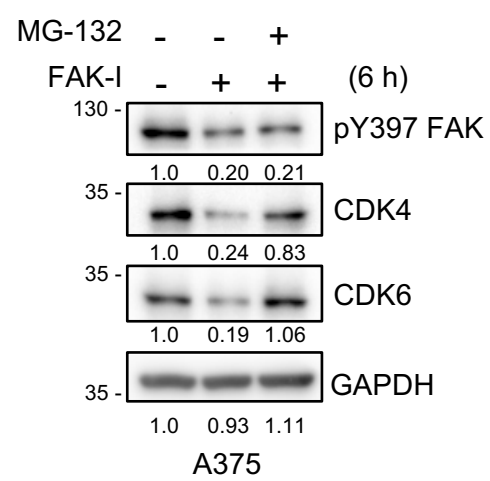

**B**

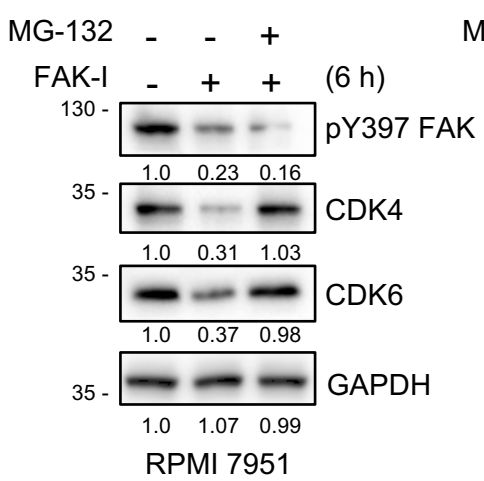

**C**

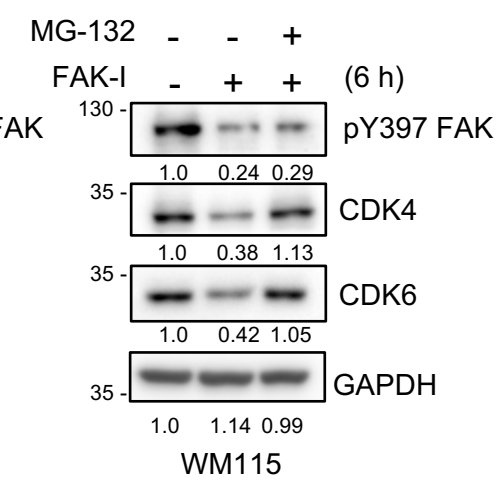

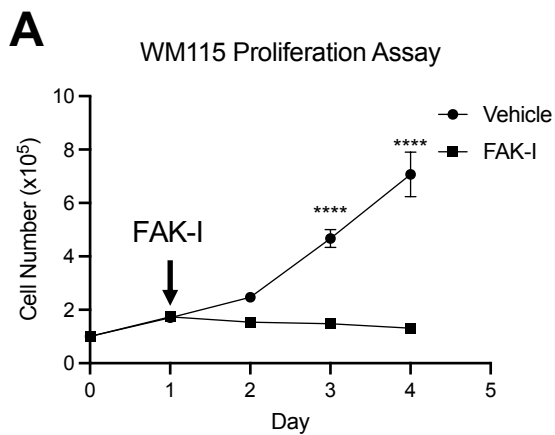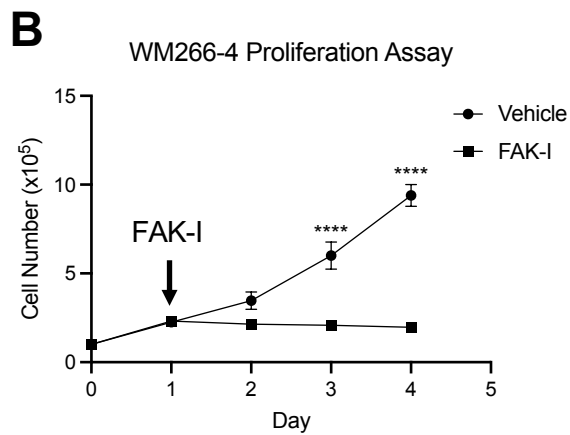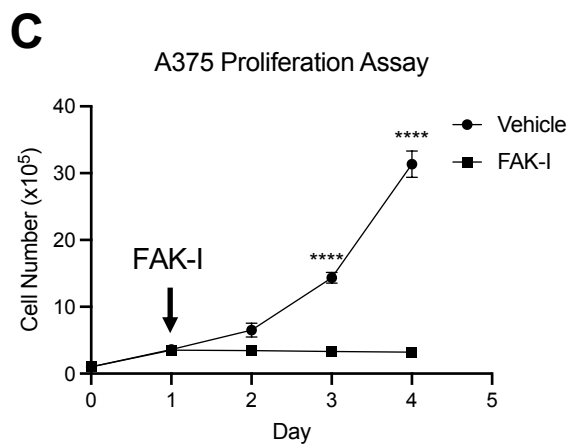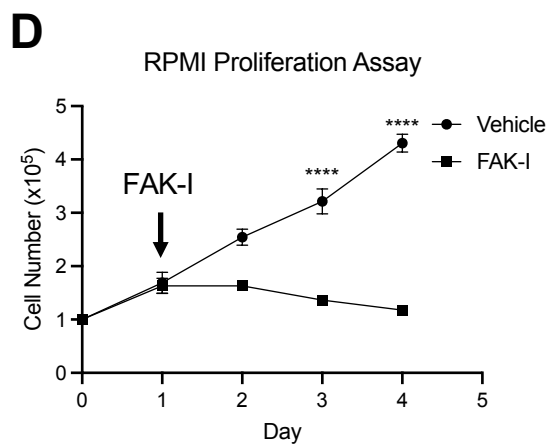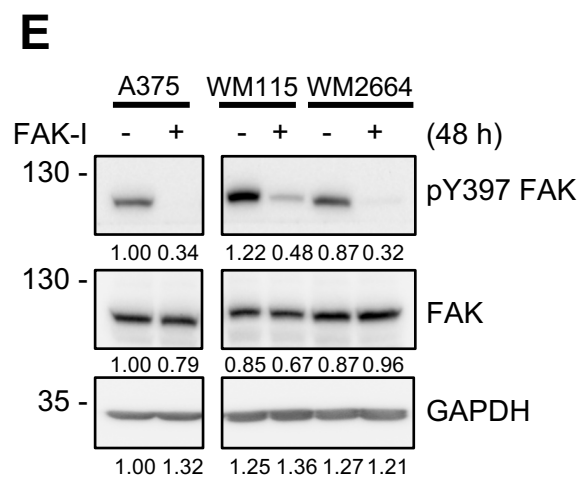

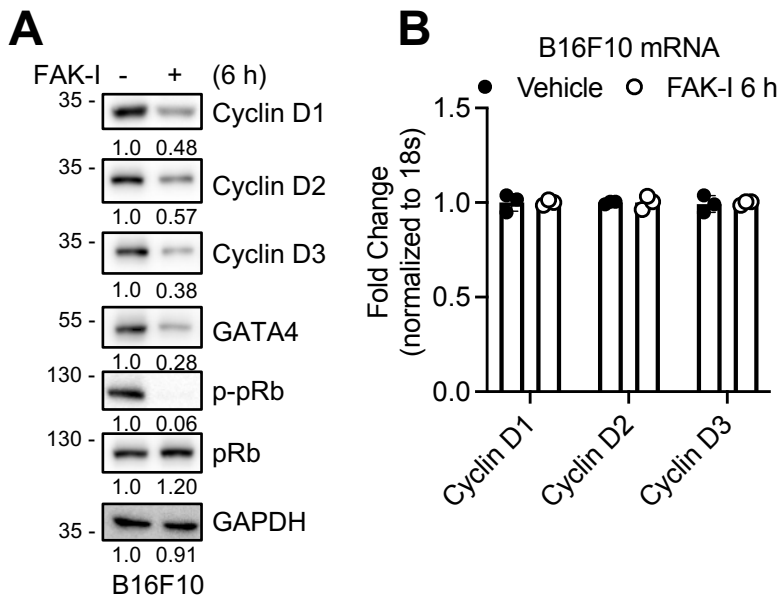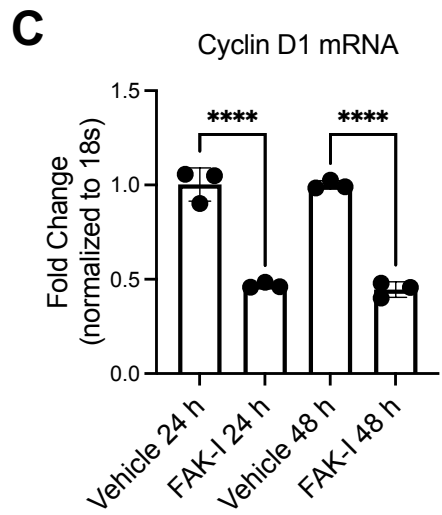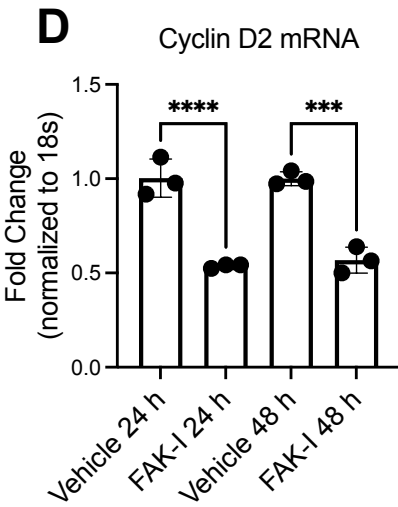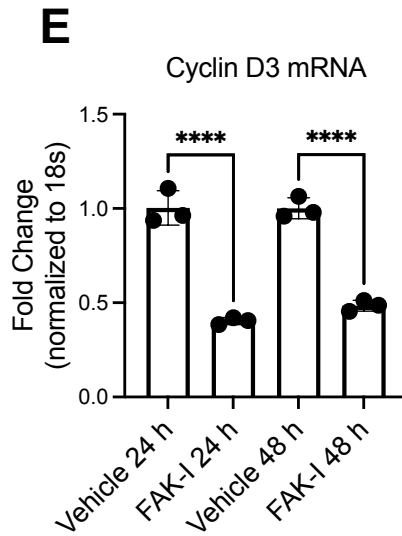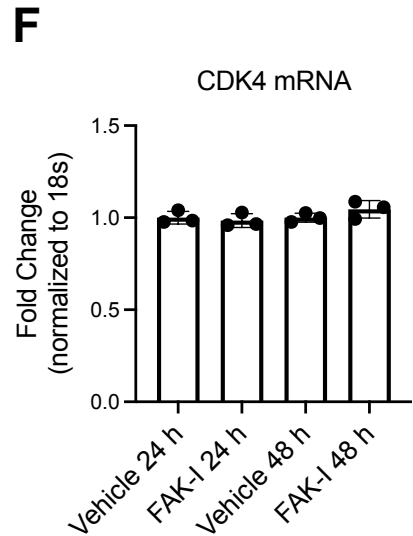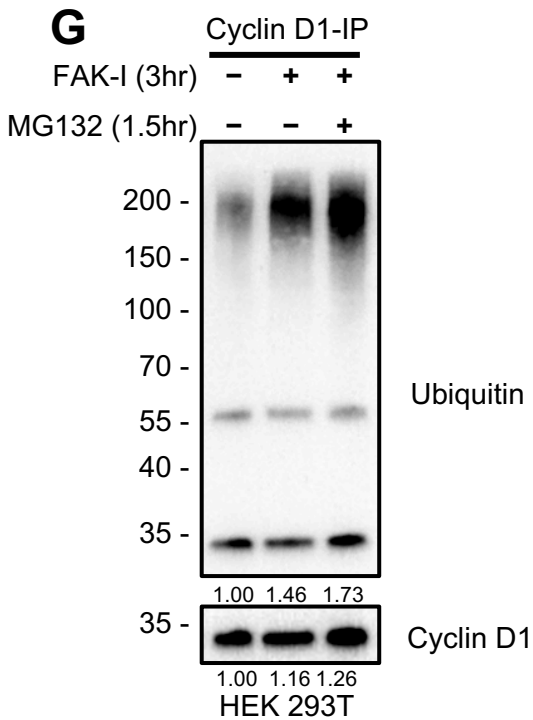

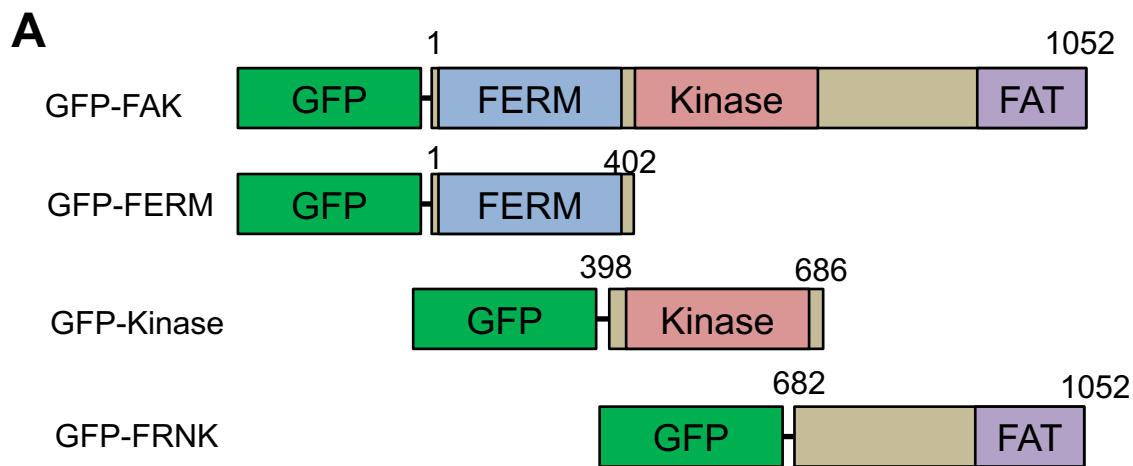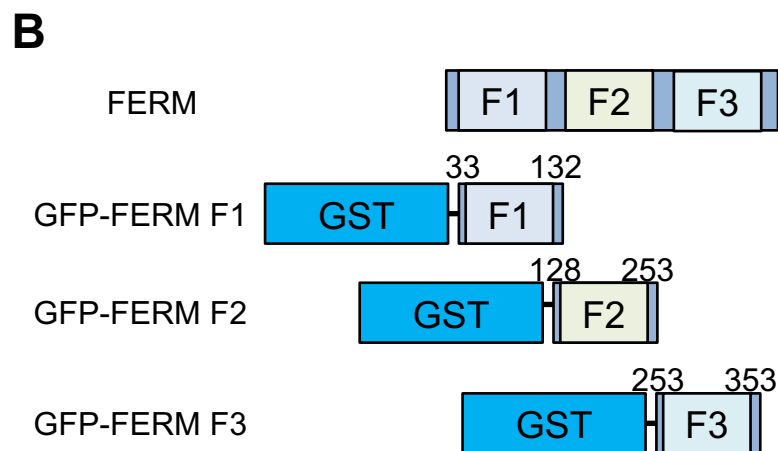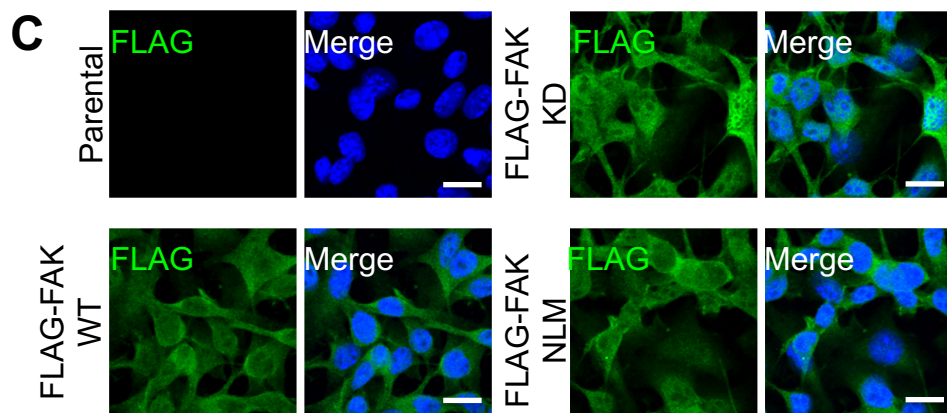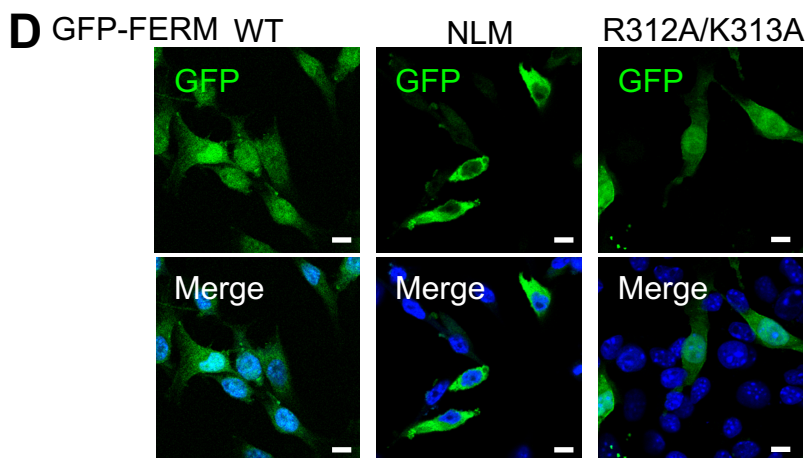

**A**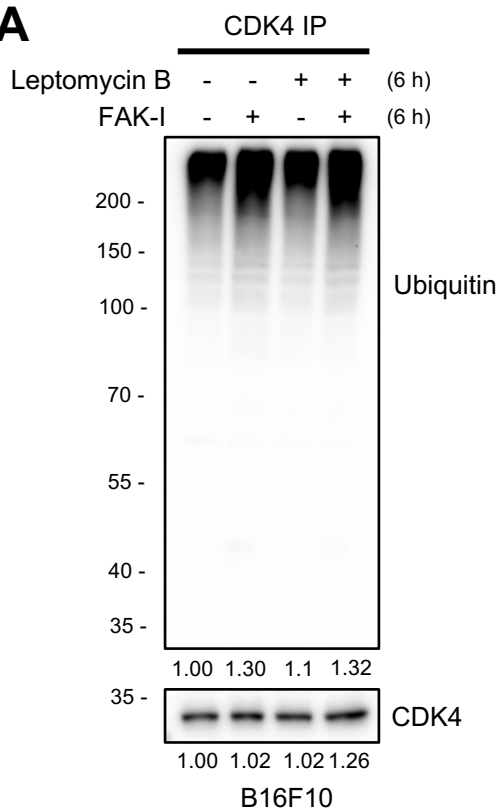**B**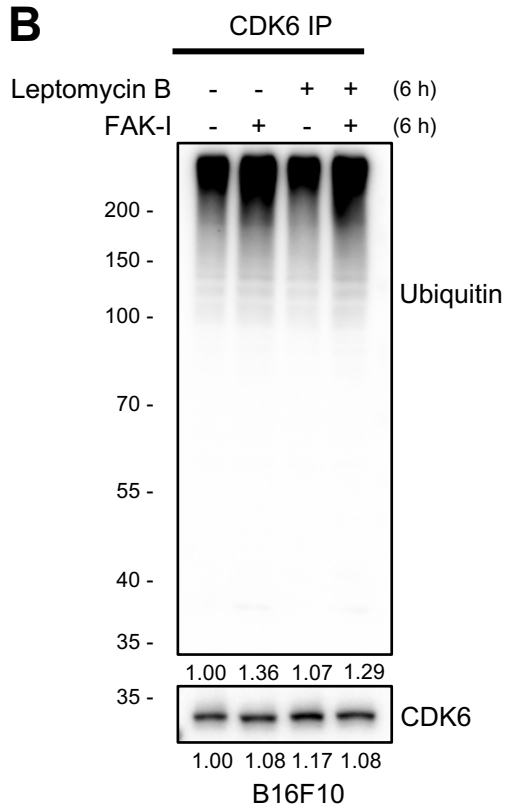

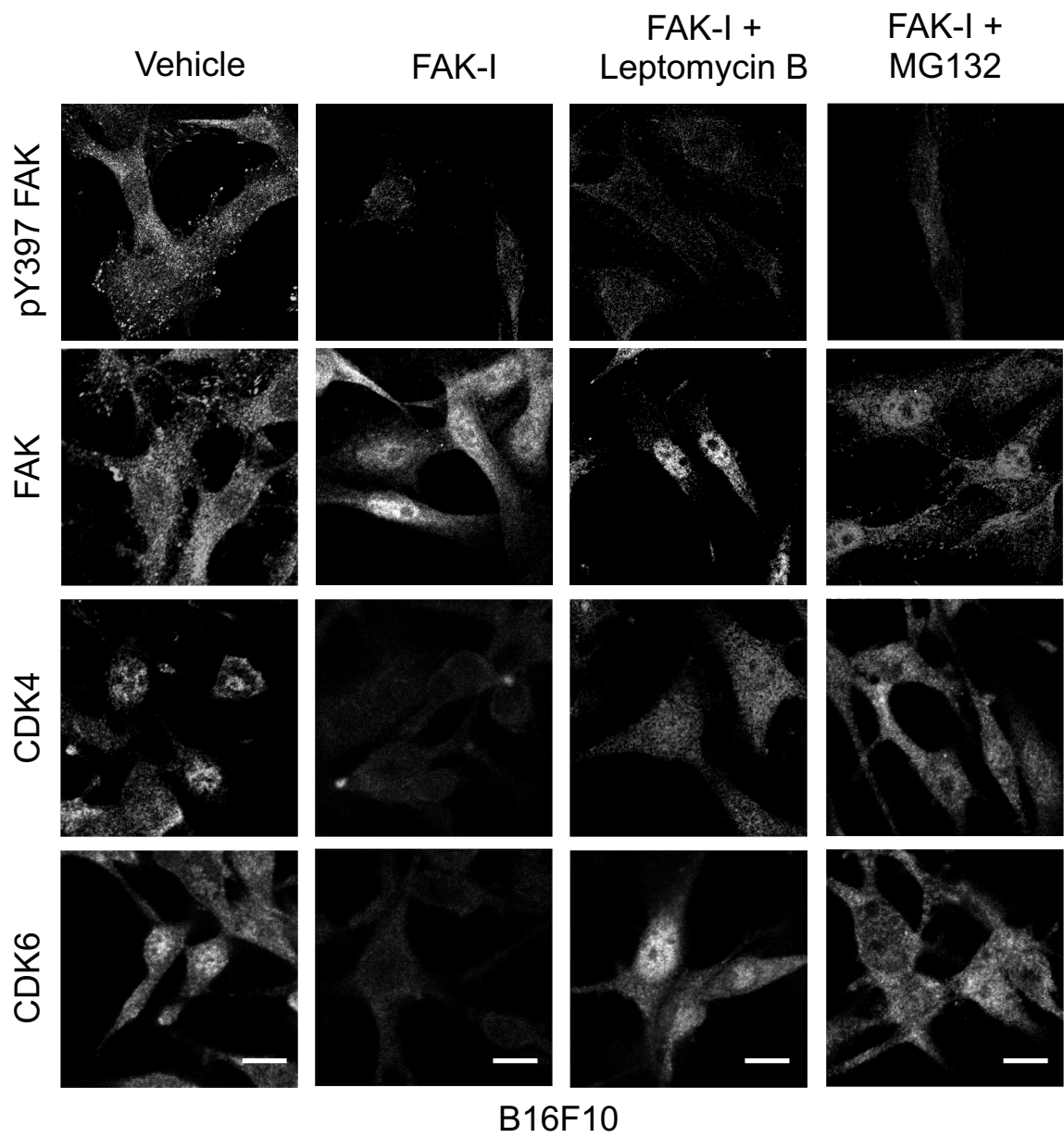

**A**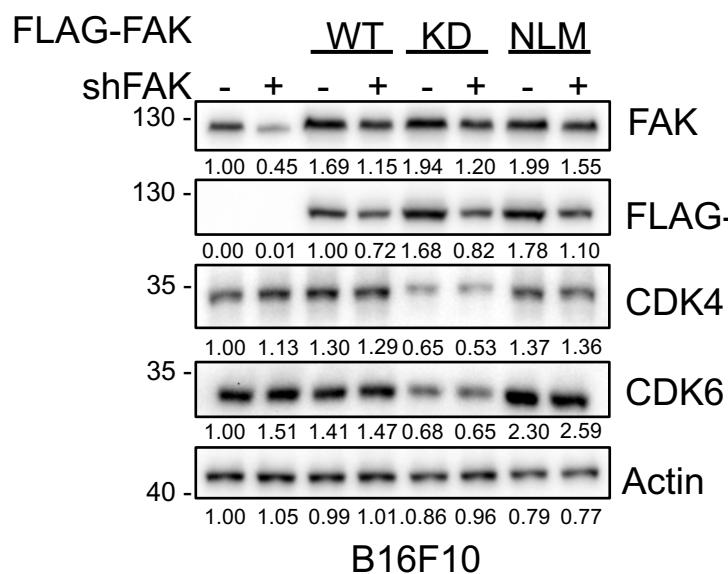**B**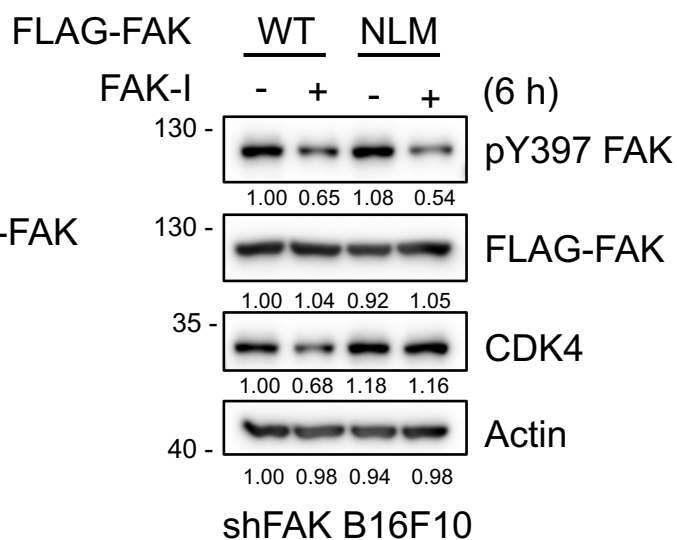**C**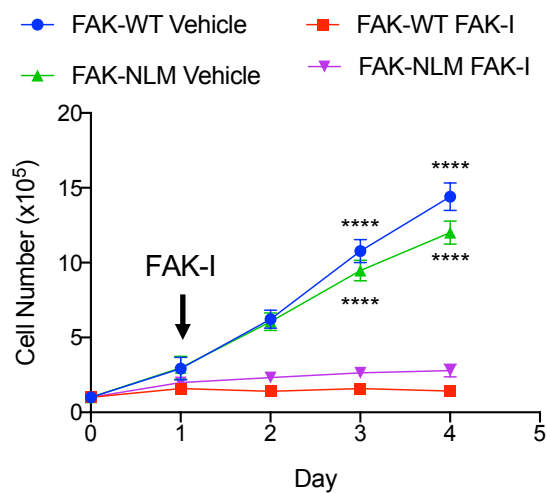**D**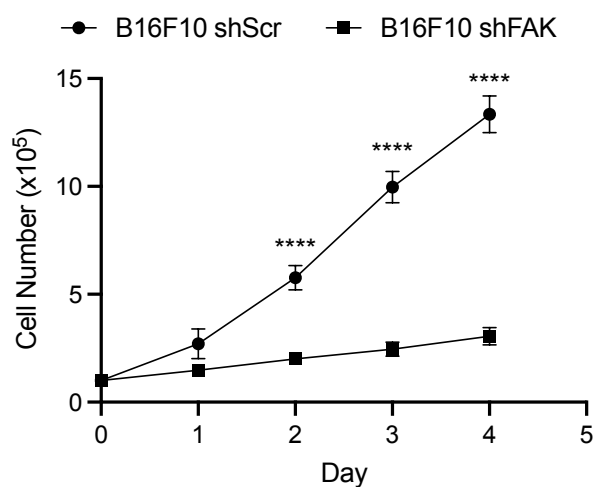

**A**

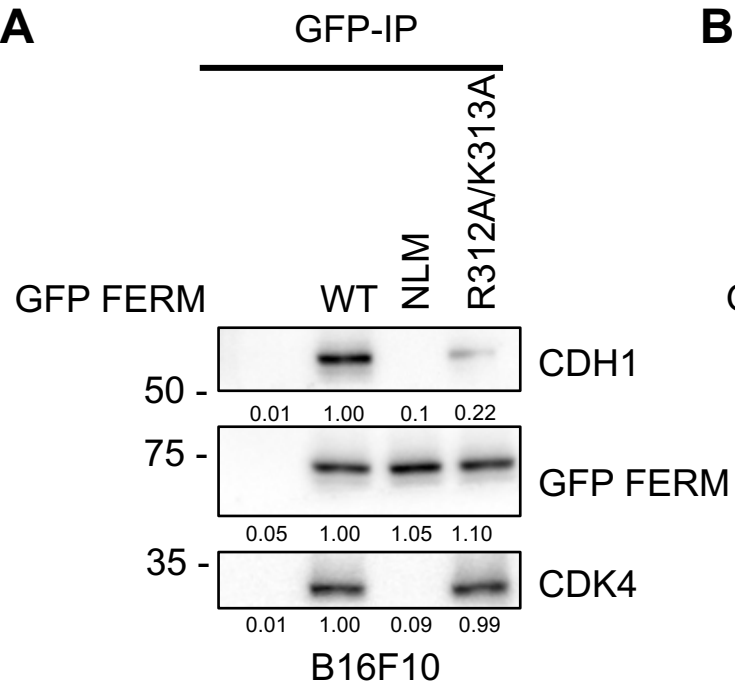

**B**

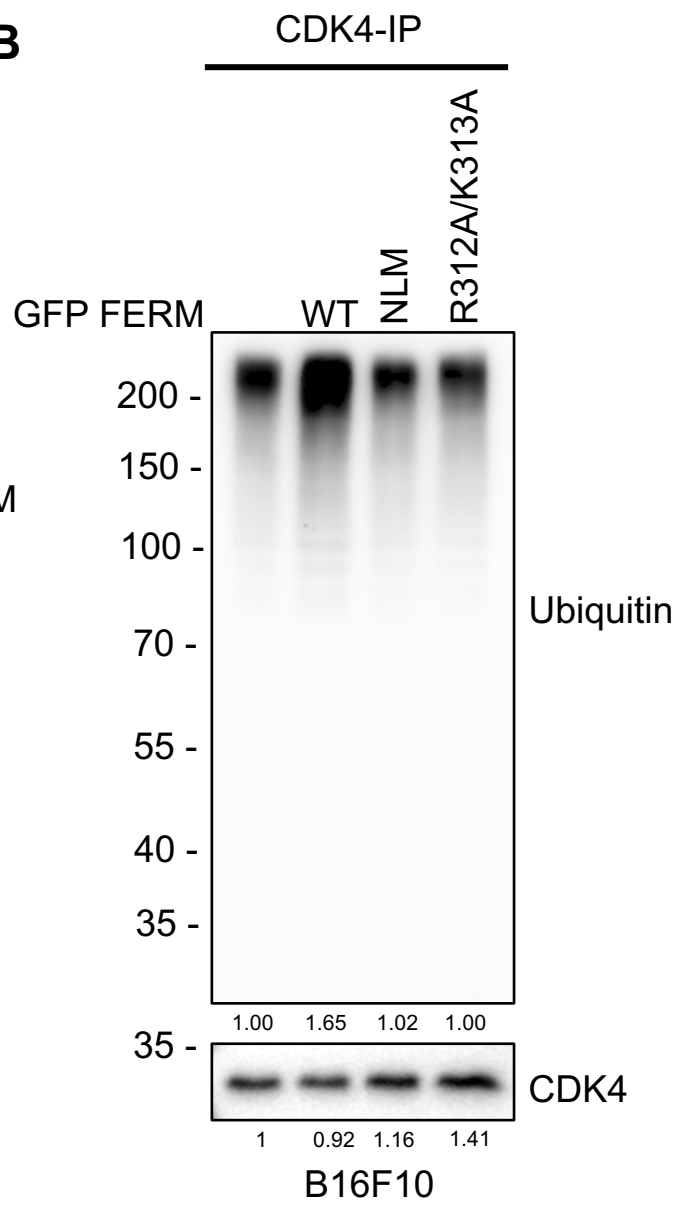

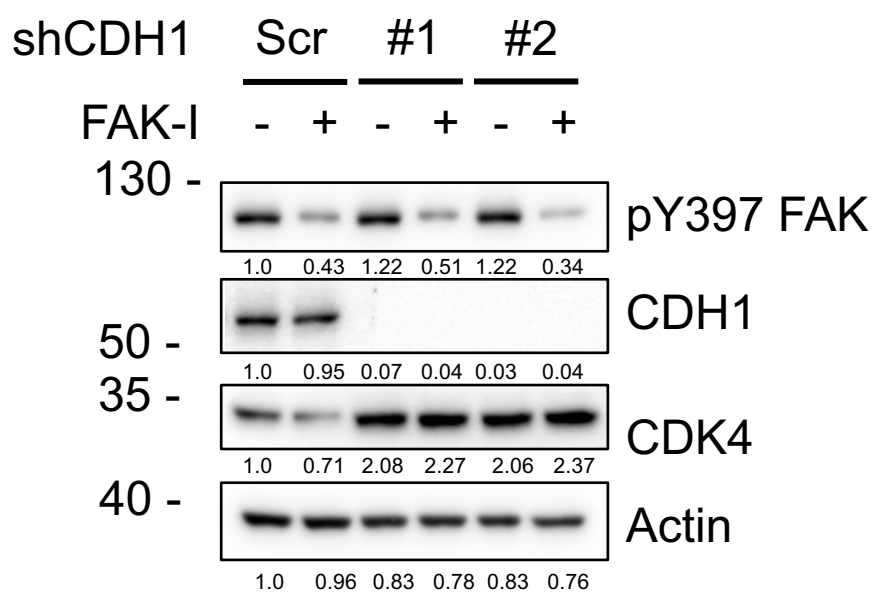

B16F10

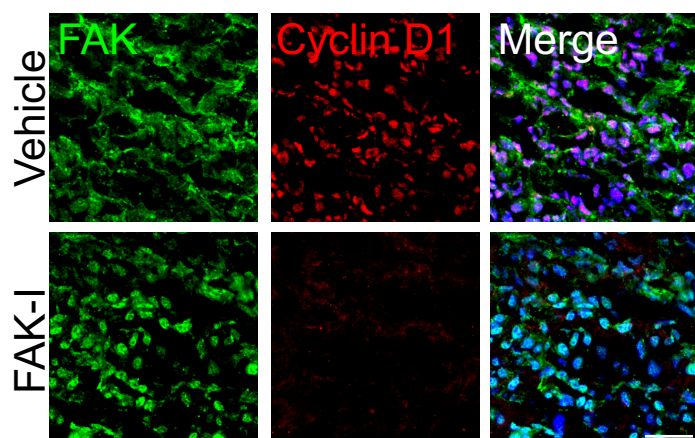

B16F10 Tumor

Normal Skin

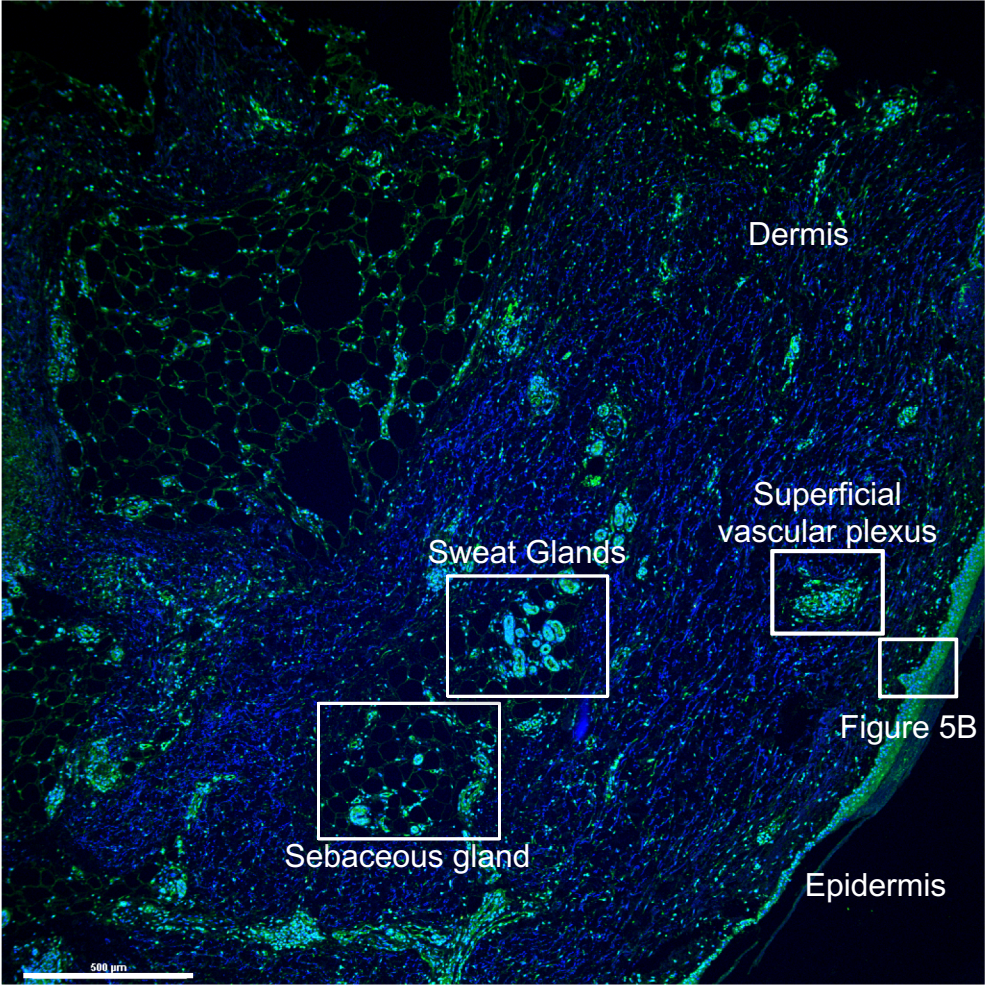

4x

**A**

Melanoma

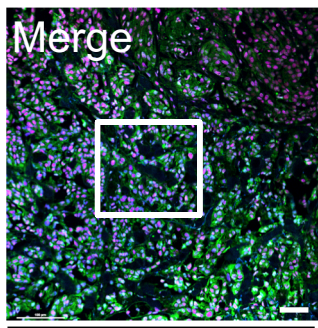

20x

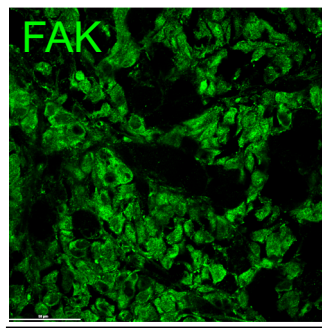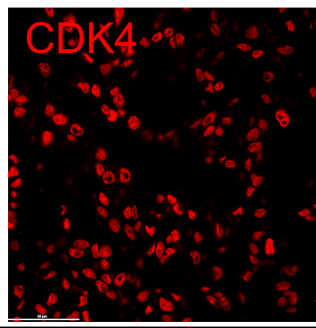

60x

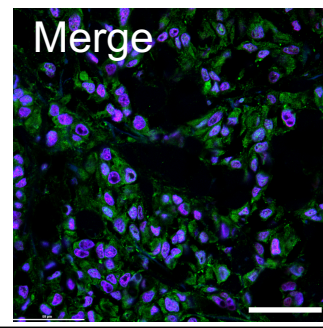**B**

Melanoma

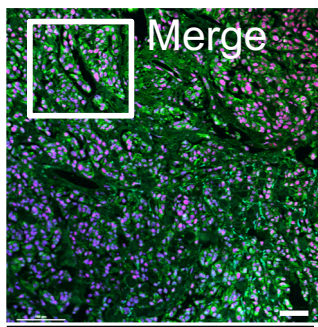

20x

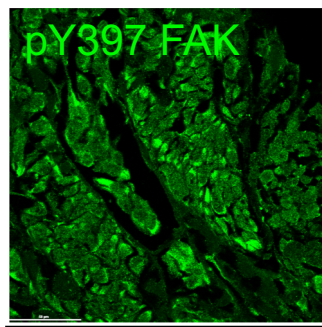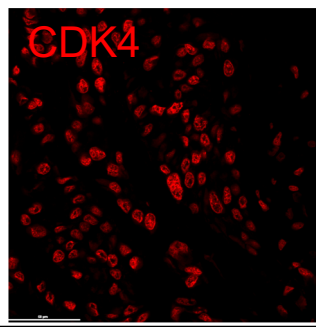

60x

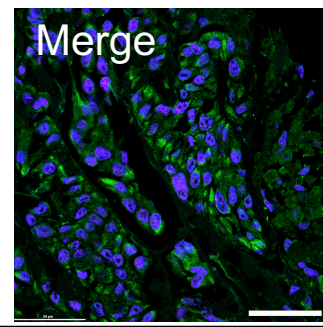

Supplement: Supporting Data 1–12 [file mmc2.pdf]
